# Engineering bone tissue with mRNA: from molecular design and delivery to clinical applications

**DOI:** 10.1016/j.mattod.2025.11.002

**Published:** 2025-12-05

**Authors:** Claudia Del Toro Runzer, Martijn van Griensven, Elizabeth Rosado Balmayor

**Affiliations:** a Department of Cell Biology-Inspired Tissue Engineering, MERLN Institute for Technology-Inspired Regenerative Medicine, Maastricht University, Universiteitssingel 40, 6229 ER Maastricht, the Netherlands; b Musculoskeletal Gene Therapy Laboratory, Rehabilitation Medicine Research Center, Mayo Clinic, Rochester, MN 55905, USA; c Experimental Orthopaedics and Trauma Surgery, Department of Orthopaedic, Trauma, and Reconstructive Surgery, RWTH Aachen University Hospital, 52074 Aachen, Germany

**Keywords:** Chemically modified mRNA, Bone regeneration, Fractures, Transcript therapy, Gene delivery, mRNA therapeutics, Tissue engineering

## Abstract

Bone possesses an intrinsic ability to regenerate after injury, but this capacity is often compromised in pathological conditions. Non-healing fractures are typically treated with autografts, which involve additional surgeries, patient discomfort, and risk of complications. Alternative strategies, such as the administration of growth factor proteins or plasmid DNA, have shown promise but are limited by high costs, immunogenicity, and safety concerns. Recently, messenger RNA (mRNA) therapies have emerged as a compelling alternative for inducing bone regeneration. Unlike DNA, mRNA functions in the cytoplasm, eliminating the need for nuclear entry and minimizing the risk of insertional mutagenesis. It is also transiently expressed and fully degradable, offering a favorable safety profile. Chemical modifications to mRNA can improve its stability, translational efficiency, and reduce innate immune activation, making it a versatile and potent tool for therapeutic applications. In this review, we explore the types of chemical modifications used to enhance mRNA performance, the delivery strategies employed for efficient cellular uptake (including *in vivo* and *ex vivo* routes), and the design of biomaterial scaffolds that support bone repair while enabling spatial and temporal control of gene expression. We also discuss the translational potential of mRNA-based approaches, including safety considerations, manufacturing scalability, and cost-effectiveness. Collectively, these advances position chemically modified mRNA as a next-generation therapeutic for bone regeneration, with the potential to overcome the limitations of current treatments and improve outcomes for patients with challenging fractures.

## Introduction

Bone is a dynamic tissue that undergoes continuous remodeling throughout life, balancing osteoclast-mediated resorption and osteoblast-mediated formation to maintain strength and mineral homeostasis [1]. This intrinsic regenerative capacity enables most fractures to heal spontaneously within 6–8 weeks [2]. However, in complex pathological conditions such as osteoporosis, Paget’s disease, osteogenesis imperfecta, or bone tumors, as well as in cases of infection, diabetes, or poor vascularization, the fracture healing process is impaired [3]. These patients face an increased risk of delayed unions or nonunions, which remain a major orthopedic challenge worldwide [4].

Current clinical strategies are limited. Autologous bone grafting, the gold standard, is constrained by donor-site morbidity, limited tissue availability, and patient discomfort [5,6]. Alternatives such as allografts and xenografts carry risks of immune rejection and disease transmission [6,7], while biomaterials such as calcium phosphate ceramics or bioactive glass lack sufficient mechanical strength for large defects [8,9]. Biologic approaches, including cell therapies and recombinant growth factors, have broadened the treatment landscape but face their own hurdles. Cell-based therapies lack intrinsic osteoconductivity and biomechanical stability [10–12], while recombinant proteins require supraphysiological doses, are expensive to manufacture, and have been associated with inflammation, ectopic bone formation, and even potential carcinogenicity [13–15]. These limitations underscore the need for safer, more effective, and more affordable therapeutic strategies.

Transcript therapy, which relies on the delivery of exogenous mRNA to drive local protein synthesis, has emerged as a compelling alternative. Unlike recombinant proteins, transcript therapy enables the *in situ* production of osteogenic factors with native conformation and post-translational modifications, offering both biological and economic advantages. As a therapeutic agent mRNA seems more attractive over DNA from a safety perspective, it does not require nuclear entry and poses no risk of genomic integration [16]. Moreover, mRNA can be rapidly and cost-effectively produced using cell-free systems [17,18].

**Fig. 1.**
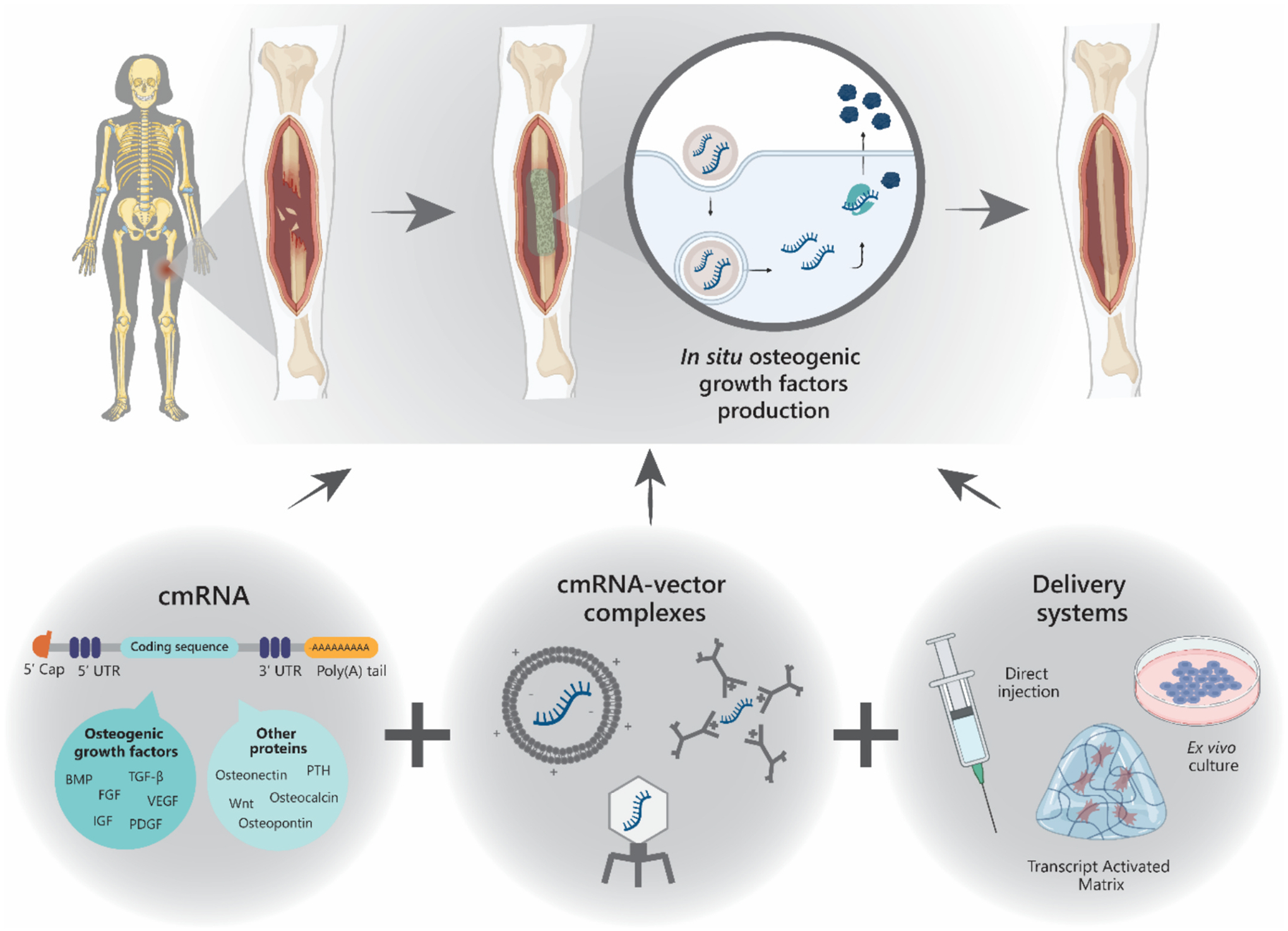
cmRNA-based strategies to enhance bone regeneration. cmRNA enables the localized production of osteogenic proteins directly at the defect site. Structural modifications to the mRNA molecule increase its stability and translational efficiency, while appropriate delivery vectors ensure protection and targeted release. In combination with optimized delivery systems, cmRNA offers a safe, cost-effective, and reliable alternative to conventional protein- and gene-based therapies, making it particularly promising for challenging or delayed fracture healing.

Nevertheless, unmodified (naked) mRNA is highly unstable, has poor translational efficiency, and is prone to activating innate immune sensors [19]. These drawbacks have motivated the development of chemically modified mRNA (cmRNA), in which optimized 5′ caps, poly(A) tails, untranslated regions, and nucleoside substitutions collectively improve stability, reduce immunogenicity, and enhance protein expression [20]. While numerous reviews have discussed mRNA therapeutics in oncology and vaccines, a dedicated focus on cmRNA for bone regeneration is lacking (Fig. 1). This comprehensive review addresses that gap by (i) summarizing the state of the art in cmRNA design and delivery strategies, (ii) highlighting *in vitro* and *in vivo* studies using cmRNA to promote bone repair, (iii) discussing translational opportunities and challenges, and (iv) assessing affordability and safety in comparison with protein- and DNA-based therapies. In the following section, we first introduce transcript therapy as a general strategy for bone regeneration, before turning to the unique features, components, and implications of mRNA-based approaches.

## Transcript therapy for bone regeneration

The absence of robust and feasible options to enhance the healing of nonunions or delayed unions has driven research toward biological therapies. These strategies focus on manipulating protein expression, either through the direct delivery of recombinant proteins or via gene therapy. Both approaches ultimately supply osteoinductive proteins, such as growth factors and transcription factors, to stimulate bone repair [21].

Recombinant proteins have demonstrated clinical benefits, but their use often requires supraphysiological doses to achieve therapeutic efficacy, which increases the risk of adverse effects [22,23]. Their high production costs further limit clinical application [24]. These challenges spurred interest in gene therapy, which delivers nucleic acids into cells to enable sustained synthesis of one or more proteins at the injury site, with native conformation and proper post-translational modifications [25]. Compared with recombinant proteins manufactured in prokaryotic systems, *in vivo* protein synthesis generally produces superior biological effects [26]. While mammalian expression systems (e.g., CHO cells) yield proteins with more accurate modifications [27], they still generate heterogeneity in glycosylation patterns that can compromise stability and function [28], and their relatively low yields (~5–10 g/L) contribute to high production costs [29]. Importantly, gene therapy also enables delivery of transcription factors, signaling molecules, and receptor proteins to their physiological cellular compartments, offering advantages over recombinant protein delivery [30].

Plasmid DNA (pDNA) was the first nucleic acid explored for fracture healing. Compared to recombinant proteins, pDNA provides greater efficiency, as a single molecule can drive protein production for hours to days, while maintaining post-translational modifications and targeting proteins to specific compartments. However, pDNA use is hampered by safety concerns, since delivery often requires viral vectors, which carry risks of random genomic integration. Moreover, expression from pDNA can result in aberrant mRNA isoforms [31].

Messenger RNA (mRNA) has since emerged as a promising alternative, offering improved safety and efficiency. Unlike pDNA, mRNA does not integrate into the host genome, eliminating the risk of insertional mutagenesis. Its transient nature limits the chance of prolonged or uncontrolled expression [32], and because it is translated directly in the cytoplasm, mRNA is effective even in non-dividing cells [33].

mRNA therapy enables the localized delivery of osteoinductive proteins at physiologically relevant levels to accelerate fracture healing [34,35]. In selecting candidate therapeutic genes for mRNA-based bone regeneration, an important consideration is their prior clinical use and regulatory approval. For instance, bone morphogenetic proteins (BMPs) −2 and −7 and platelet-derived growth factors (PDGFs) have already received FDA approval for certain orthopedic applications [36–38], while vascular endothelial growth factor (VEGF) has been evaluated in multiple clinical trials [39,40]. Leveraging growth factors with established safety and efficacy profiles can significantly expedite translation of mRNA-based approaches to the clinic, compared to factors that remain untested in humans.

Beyond these clinically established growth factors, there is also strong rationale to investigate additional targets for bone regeneration, even though they have not yet reached FDA approval. cmRNAs encoding molecules such as fibroblast growth factors (FGFs), transforming growth factor-β1 (TGF-β1), insulin-like growth factors (IGFs), osteonectin, osteopontin, or Wnt signaling proteins have already shown promising regenerative effects [35,41–43]. While clinical translation of these targets may take longer due to the absence of regulatory approval, their study remains valuable for uncovering new biological mechanisms and potentially identifying synergistic effects. In particular, combining such novel factors with well-characterized growth factors like BMP-2 or VEGF could amplify or fine-tune the regenerative cascade, offering more physiological and sustained outcomes. Thus, both established and emerging therapeutic genes should be considered in the design of next-generation mRNA-based strategies for bone healing. To date, only cmRNAs encoding BMPs, VEGF, Runx2, and AMELX have been evaluated in bone regeneration studies *in vitro* and *in vivo* (Table 1).

Another critical point is whether transient expression is sufficient to promote bone healing. Preclinical and clinical studies with recombinant proteins have demonstrated that a single local administration of potent growth factors, such as BMP-2, BMP-7, or VEGF, can effectively trigger fracture repair, despite the process involving the coordinated activity of numerous signaling molecules [21,24]. This is possible because these factors act as master regulators, activating downstream pathways that sustain osteogenesis or angiogenesis long after their direct presence has diminished. However, achieving therapeutic effects with recombinant proteins typically requires very high doses, which lead to severe side-effects. In contrast, cmRNA-based approaches may benefit from brief (e.g., one to two weeks) sustained expression at lower doses, providing sufficient biological activity during the critical early phase of bone repair [44,45]. Taken together, the choice of therapeutic gene depends not only on its biological role in bone repair, but also on its regulatory status and the temporal dynamics of its activity.

Despite its therapeutic promise, unmodified (naked) mRNA is inherently unstable and immunostimulatory [16]. To address these limitations, chemically modified mRNAs (cmRNAs) have been developed. Key modifications include optimized 5′ caps (e.g., anti-reverse cap analogs), poly(A) tails, engineered 5′ and 3′ untranslated regions (UTRs), and partial or complete substitution of immunogenic nucleotides, such as cytidine and uridine [20]. These modifications enhance stability, reduce innate immune activation, and improve translational efficiency, establishing cmRNA as a promising therapeutic platform for fracture healing.

## mRNA modification

**Fig. 2.**
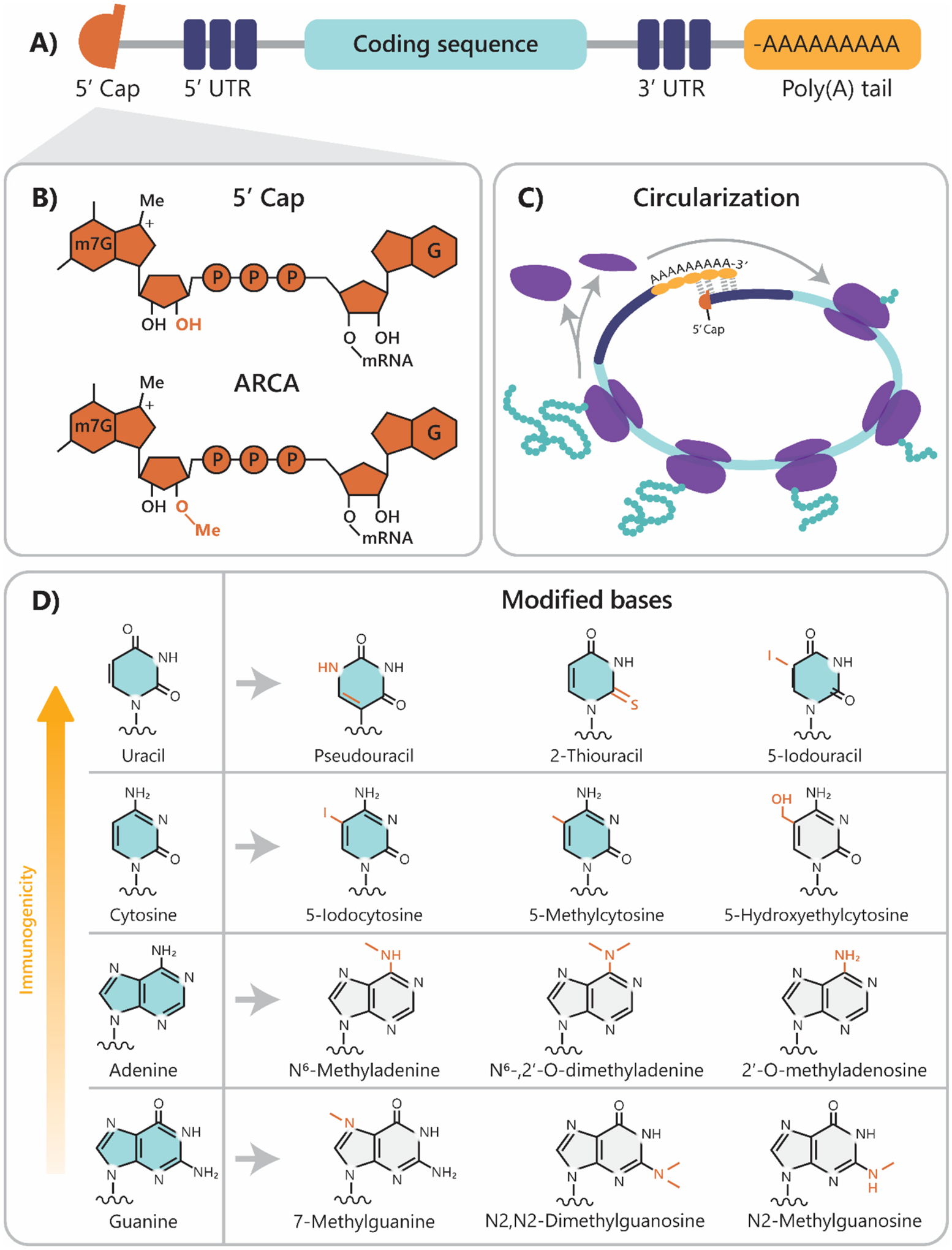
Structural elements of mRNA and commonly used chemical modifications. (A) Native mRNA is composed of five key structural elements: the 5′ cap, 5′ untranslated region (UTR), coding region, 3′ UTR, and poly(A) tail. (B) The 5′ cap plays a central role in protecting mRNA from degradation and initiating translation. The anti-reverse cap analog (ARCA) is commonly used in cmRNA synthesis to ensure correct cap orientation and to reduce innate immune activation during *in vitro* transcription. (C) The poly(A) tail enhances mRNA stability and translation. Through its interaction with the 5′ cap and poly(A)-binding protein (PABP), it promotes mRNA circularization, ribosome recycling, and protection against exonucleolytic decay. (D) Common base modifications used in cmRNA to evade immune detection by Toll-like receptors (TLRs), RIG-I, and other immune sensors. Bases and their modified analogs that have been applied specifically in cmRNA-based bone regeneration studies are highlighted in light blue. Several modifications for each base are possible as indicated by the examples following the arrow.

Native mRNA is a single-stranded polynucleotide complementary to a DNA template strand. It functions as an intermediate that transfers genetic information from the nucleus to the cytoplasm, where it serves as a template for protein synthesis. Structurally, mRNA consists of five key elements: the 5′ cap, 5′ and 3′ UTRs, the coding region, and the poly (A) tail (Fig. 2A). When designing cmRNA, each of these elements must be included and can be optimized to improve protein yield, stability, and translational efficiency [51].

### The 5′-cap

The 5′ cap, or 7-methylguanosine cap (m7G), is a modified guanine nucleotide added post-transcriptionally to the first nucleotide of the premRNA via a 5′–5′ triphosphate bridge (m7GpppN) (Fig. 2B) [52]. This cap is essential for mRNA stability, efficient translation, nuclear export, splicing, and polyadenylation. Most eukaryotic translation is cap-dependent: the cap structure recruits the eukaryotic translation initiation factor 4F (eIF4F) complex, which in turn facilitates the assembly of the small ribosomal subunit to initiate translation [53].

The unique structure of the 5ʹ-cap sterically protects the mRNA from exonuclease degradation (e.g., by Xrn1p) [54] and plays a critical role in immune evasion [32]. There are numerous RNA based immune sentinels, some of which sensor the presence of the 5′-cap to distinguish between self vs. non-self RNA. The cytosolic innate immune receptor Retinoic Acid Inducible Gene-I (RIG-I) is the principal detector of pathogenic and synthetic mRNAs lacking 5′-end capping [55]. Similarly, the melanoma differentiation associated gene 5 (MDA5), detects long double stranded RNA lacking of 5′-end capping [56]. Upon activation, both RIG-I and MDA5 trigger a type I interferons (IFNs) response [32]. Proper capping therefore reduces the risk of immune activation upon exogenous cmRNA delivery.

There are two main approaches to generate capped mRNA: (1) enzymatic capping using recombinant vaccinia virus-derived enzymes, and (2) co-transcriptional capping with cap analogs during *in vitro* transcription. The enzymatic capping process allows a complete capping of the 5′ ends of mRNA, so it can be used in large-scale production of mRNAs or at laboratory scale by ready-to-use capping enzyme kits [51]. The resulting cap structure is limited to the standard cap structure (m7GpppG), and the process requires several additional steps after the *in vitro* synthesis of mRNA. These include hydrolysis of the 5′-triphosphate to a diphosphate, GMP transfer, and successive methylation steps [57].

In contrast, co-transcriptional capping is simpler, cost-effective, and allows for incorporation of diverse cap analogs (e.g., ${\text{m}}_{2}^{7,2\prime \;\text{-O}}{\text{Gp}}_{3}\text{G}$, m^7^2′dGp_4_G, and ${\text{m}}_{2}^{7,3\prime \text{-O}}{\text{Gp}}_{5}\text{G}$) [58]. However, the main limitation of this technique is that the synthetic cap analogue and the GTP nucleotide required for *in vitro* transcription compete each other, resulting in some of the mRNA remaining uncapped and inactive [33]. Moreover, cap analogs can be incorporated on a functional forward orientation (m7GpppG), or in a reverse orientation (Gpppm7G), which is unable to support initiation of translation [57]. To address this, anti-reverse cap analogs (ARCA), which are methylated or deoxygenated at the 3′–OH of the N7-methylguanosine ribose (${\text{m}}_{2}^{7,3\prime \text{-O}}\text{GpppG}$ or m ^7,3′-d^ GpppG), were developed to ensure only forward incorporation (see Fig. 2B) [57]. More recently, ARCAs with modified triphosphate linkages (e.g., sulfur, borane, selenium) have been introduced to further enhance resistance to decapping enzymes while generating perfectly forward-oriented mRNAs [59].

A major advancement in co-transcriptional capping has been the introduction of CleanCap^®^ technology, which is now considered the gold standard for therapeutic mRNA production. CleanCap^®^ enables highly efficient, one-step incorporation of a cap1 structure (m7GpppNm) during *in vitro* transcription, eliminating the issue of reverse incorporation while achieving > 95 % capping efficiency [60]. Compared to traditional ARCA-based approaches, CleanCap^®^ generates transcripts with improved translational efficiency, reduced immunogenicity, and batch-to-batch consistency, making it particularly attractive for clinical manufacturing [61]. Further developments have expanded capping options to cap2 structures (m7GpppNmNm), which better mimic native mammalian mRNAs and show reduced innate immune recognition [62]. Additionally, incorporation of N6-methyladenosine (6 mA) at the first transcribed nucleotide has been reported to enhance translation and stability [63], representing another layer of fine-tuning in cap engineering.

Beyond canonical cap structures, more advanced chemotopological approaches have been developed. For instance, Chen et al. introduced the ligation-enabled mRNA–oligonucleotide assembly (LEGO) platform, which uses branched, dual-capped motifs to augment translation capacity in both linear and circular mRNAs. This approach, enhanced eIF4E–eIF4G binding, improved stability against decapping, and achieved up to tenfold higher protein production *in vivo* [64]. Similarly, structural studies on phosphorothioate-containing cap analogs have revealed how O-to-S substitutions within the β-phosphate (β-S-ARCA diastereomers D1 and D2) stabilize binding to eIF4E through electrostatic “thio-effects.” Crystallographic analyses demonstrated that sulfur or selenium substitutions enhance electrostatic interactions with positively charged residues, explaining the superior translational properties of these analogs and supporting their clinical use in mRNA vaccines [65].

In bone-targeted mRNA therapies, the most commonly used methods are enzymatic capping and co-transcription with standard ARCA analogs [5]. However, the field could substantially benefit from the integration of these next-generation capping strategies, which could potentially accelerate the transition from preclinical proof-of-concept studies to clinically viable mRNA therapies.

### Poly(A) tail

At the opposite extreme of the mRNA strand the 3ʹ poly(A) tail is situated, an essential domain that controls mRNA stability. As its name suggests, it is made by a chain of adenosine nucleotides with a length that varies depending on the cell type, for HeLa and NIH 3T3 cells it is of 50–100 nucleotides. The poly(A) tail protects the mRNA from 3′ to 5′ nuclease degradation by binding to the polyadenylate-binding protein 1 (PABP). Despite being at the end of the molecule, the poly(A) tail is required for translation initiation. When the PABP binds the poly(A) tail, it then interacts with eIF4G, forming a pre-initiation complex together with the 5′-cap. This synergistic effect of the cap structure and the poly (A) tail forms a close loop (see Fig. 2C) or mRNA circularization, which facilitates the recycling of ribosomes while protecting from exonucleolytic degradation, thus yielding superior translation over linear mRNA [59]. Furthermore, the circularization of mRNA bypass cellular RNA sensors and thereby avoid triggering an immune response [66].

Similar to the 5′-cap, the poly(A) tail can be added using two approaches: (1) enzymatically, with recombinant poly(A) polymerase, or (2) by encoding the poly(A) in the template vector from which it is transcribed [5]. The enzymatic method allows the incorporation of modified nucleotides but results in heterogeneous tail lengths. Poly(A) length control is important because it acts as a timer of mRNA stability and it affects translation efficiency, mRNAs with longer tails are translated better than mRNAs with short tails [67].

By contrast, *in vitro* transcription of RNA requires a poly(T) containing DNA template. This process yields RNA with a defined poly(A) tail length, and is therefore preferred, especially for clinical applications where variations in the tail size can either promote or retard mRNA decay, therefore affecting the mRNA half-life [68]. A disadvantage of using this method is that plasmids containing poly(A) regions recombine in *E. coli*, resulting in extensive shortening of the poly(A) tail. A solution proposed by Trepotec et al. involves using segmented poly(A) tails consisting of two or more poly(A) blocks (40–60 nt) separated by spacer sequences to prevent recombination without compromising mRNA performance [69].

While the optimal tail length may vary by cell type, ~120 adenosines is generally considered ideal [25]. In the field of cmRNA for bone regeneration, most of the studies report the use of the poly(A) tail encoded in the template vector of a length of 120 [41] or 200 nucleotides [70]. Two groups have used enzymatic polyadenylation of 120 [71], and 200 nucleotides [72].

Recent advances have explored not only length but also chemical and topological modifications of the poly(A) tail to improve mRNA performance. A study on branched chemically modified poly(A) tails demonstrated that the introduction of multiple synthetic poly(A) tails markedly enhances translation capacity, yielding ~ 4.7–19.5-fold higher protein expression *in vitro* and extending *in vivo* expression from <7 days to 14 days [73]. This strategy also enabled efficient multiplexed genome editing of clinically relevant targets at minimal mRNA dosages, underscoring its potential as a broadly applicable design principle for therapeutic mRNAs.

### 5′ and 3′ UTRs

Flanking the coding sequence of mRNA are the 5′ and 3′ UTRs, which contain regulatory elements that play central roles in transcript stability and translational efficiency. Although they do not encode protein, UTRs harbor sequence motifs and structural features that recruit RNA-binding proteins or modulate ribosome engagement, thereby fine-tuning protein production [74].

One major strategy has been the use of natural UTRs derived from highly stable endogenous mRNAs, such as those of the human α- and β-globin genes. These UTRs contain pyrimidine-rich elements that enhance poly(A)-tail–PABP interactions and confer resistance to exonucleolytic decay, extending transcript half-life [75]. Their clinical relevance is underscored by their inclusion in the approved COVID-19 vaccines Comirnaty/BNT162b2 (BioNTech–Pfizer) and Spikevax/mRNA-1273 (Moderna), where they were selected after systematic screening to minimize inhibitory secondary structures, reduce innate immune activation, and optimize ribosome recruitment [76,77]. Similarly, Ferizi et al. [78] systematically tested UTRs from transcripts with naturally long half-lives. When cytochrome *b*-245 alpha chain (CYBA) UTRs were fused to BMP-2 mRNA, they yielded the highest protein expression levels and promoted osteogenic differentiation of human adipose-derived mesenchymal stem cells. These studies highlight how endogenous UTRs provide solutions for enhancing stability and expression, serving as reliable design elements for therapeutic cmRNAs in bone regeneration.

A second approach involves rational modification and synthetic engineering of UTRs to optimize expression and reduce undesired interactions. At the 5′ UTR, specific motifs regulate ribosome entry through interaction with the eIF4F complex. For instance, translation is more efficient when the start codon (AUG) resides within a Kozak consensus sequence (GCCA/GCCAUGG) [79]. Internal ribosome entry sites (IRES), often derived from viral sequences, are also commonly used to promote cap-independent initiation [51,80]. Elfakess et al. identified a unique regulatory motif known as the Translation Initiator of Short 5′ UTR (TISU), which promotes highly efficient cap-dependent initiation [81]. Building on this, another group engineered BMP-2 cmRNA incorporating a TISU element, achieving robust BMP-2 production, upregulation of osteogenic and angiogenic genes, and successful bone regeneration in a rat femoral defect model [71].

Another aspect of engineering focuses on secondary structure and sequence length. Highly structured UTRs can hinder ribosome scanning or promote binding by inhibitory microRNAs. Chemically modified nucleotides may alter these structures, sometimes reducing innate immune activation but also impairing translation [82]. To overcome these challenges, “minimalistic” UTRs of reduced length have been developed, which avoid inhibitory folding and microRNA interactions. These streamlined sequences showed equal or even superior expression compared to natural UTRs and were robust across multiple cell types [74,83]. At the 3′ UTR, AU-rich elements (AREs) have classically been associated with transcript destabilization by accelerating degradation [84]. However, recent work demonstrates that AREs do not universally promote decay; instead, their effect depends on sequence context and protein interactions. For example, optimized AREs designed to recruit the RNA-binding protein HuR were shown to enhance mRNA stability and increase protein expression up to fivefold across different transgenes [85]. Thus, while natural AREs often limit transcript half-life, rational engineering of these motifs or substitution with stabilizing elements provides an effective strategy to prolong expression, a critical requirement for bone healing.

The third and most recent category is the application of artificial intelligence (AI) and machine learning to UTR optimization. By training on large datasets of UTR sequences and their functional outcomes, AI models can predict highly effective UTR variants and generate novel sequences tailored for specific therapeutic or research applications.

Castillo-Hair et al. used deep learning on polysome-profiling data from randomized libraries to create a predictive model for 5′ UTRs across multiple cell types [86]. Their models accurately predicted UTR performance and enabled the generation of synthetic 5′ UTRs that supported robust expression of therapeutic cargos, including gene-editing enzymes. Similarly, the Optimus 5-Prime framework used convolutional neural networks trained on polysome data to predict ribosome loading and translation efficiency, allowing rational design of synthetic 5′ UTRs with tunable protein output [87]. Beyond mRNA therapeutics, it also highlighted disease-associated UTR variants that disrupt translation.

Machine learning has also been applied to 3′ UTR design. Using massively parallel reporter assays that measured the stability of over 180,000 unique sequences, researchers trained predictive models capable of guiding the synthesis of highly stable UTRs [88]. Synthetic designs extended transcript half-life, yielding up to twofold greater sustained protein production *in vitro* and dramatic increases (30–100-fold at later time points) in mouse models, compared to standard control sequences.

For bone regeneration applications, where precise control over protein dose and duration is essential, such approaches promise to complement endogenous and synthetic strategies, accelerating the development of cmRNAs with optimized therapeutic performance.

### Modified nucleotides

As cmRNA travels through the extracellular space and into the cytoplasm, it is vulnerable to recognition by innate immune receptors on both immune and non-immune cells [89]. Endogenous mRNAs are co–and post-transcriptionally modified and compartmentalized in ways that shield them from immune detection. In contrast, IVT cmRNAs, especially unmodified ones, lack these protective features and may enter endosomal compartments where they are sensed as foreign [90]. While such immune activation is desirable for vaccines, it poses a challenge for therapeutic protein production [33]. These sensing proteins are called Toll-like receptors (TLRs), and are able to trigger the secretion of IFNs, invoking a signaling cascade that causes inflammation, inhibits translation, and/or causes RNA degradation. Different types of TLR sense specific RNA structures. TLR7 and TLR8 recognizes single stranded RNA whereas TLR3 senses double stranded RNA [91,92]. Activation of some of these receptors (i.e., TLR7 and TLR8) seems unable to distinguish between foreign or self mRNA; instead, their stimulation relies on the mode of access to the endolysosomal compartment [93]. Several studies have reported that TLRs’ mechanism for detecting foreign RNA is through recognition of specific nucleotides, for example, uridine-rich and AU/GU-rich motifs are particularly immunostimulatory via TLR7/8 [94]. Therefore, to prevent recognition of synthetic mRNA, a strategy is to create ‘de-immunized’ mRNA by incorporating naturally occurring modified nucleotides (Fig. 2D). Commonly used modifications include pseudouridine (Ψ), 2-thiouridine (2TU), 5-methyluridine (5 mU), 5-methylcytidine (5mC), and N6-methyladenosine (m6A) [95]. These substitutions not only prevent recognition by TLRs but also reduce activation of cytoplasmic RNA sensors such as RIG-I and RNA-dependent protein kinase R, while generally maintaining translational competency [33]. Importantly, base modifications do not typically restrain mRNA translation by ribosomes, as they are a naturally occurring post-transcriptional process in mammalian cells [25]. For instance, pseudouridine, one of the most commonly used, is a naturally occurring modified nucleoside produced by an enzymatic isomerization of uridine by the rotation of nitrogen located at the C6 position [96]. Nonetheless, the impact of nucleotide modifications is context-dependent, outcomes vary with the type of modification, the coding sequence, and the cell type. In certain cases, specific modifications may enhance mRNA stability yet simultaneously reduce translational efficiency [97].

These modifications are typically introduced by replacing all or a percentage of native nucleotides during *in vitro* transcription. For instance, Elangovan et al. produced BMP-2 cmRNA using either a 25 % substitution with 2TU and 5mC, or 100 % substitution with Ψ and 5mC. The resulting transcripts, capped with ARCA and polyadenylated, were combined with collagen scaffolds for calvarial defect repair in rats, significantly enhancing bone regeneration [46]. Similarly, Balmayor et al. used a 25 % substitution strategy to generate stable, nonimmunogenic BMP-2 cmRNA, leading to bone formation in femoral defects within 2 weeks [35]. More recently, Zhang et al. introduced iodinated modifications by replacing 35 % of uridines with 5-iodo-pyrimidines and 7.5 % of cytidines with 5-iodo-cytidine, resulting in superior bone regeneration compared to non-coding controls [71].

### Next-generation mRNA formats

A major challenge in mRNA-based protein replacement therapies, such as those aimed at bone regeneration, is the relatively high dose requirement compared to vaccines. For instance, the COVID-19 vaccine mRNA-1273 is administered at two doses of 100 μg each, whereas clinical trials with VEGF-A cmRNA (AZD8601) have tested doses ranging from thirty 100 μg injections to as much as 1 mg [98,99]. To overcome these dose limitations, next-generation mRNA formats are being developed to enhance protein output per transcript and prolong expression. Among the most promising approaches are self-amplifying mRNAs (saRNAs) and circular RNAs (circRNAs). saRNAs incorporate viral-derived replication machinery that enables intracellular RNA amplification, thereby substantially reducing the amount of input material needed to achieve therapeutic protein levels [100,101]. circRNAs, on the other hand, adopt a covalently closed-loop structure that lacks free ends, rendering them resistant to exonuclease degradation. This enhanced stability supports sustained protein translation, making circRNAs attractive for applications requiring long-term expression [102,103].

In parallel, advances in computational design are transforming the optimization of mRNA sequences. AI-driven codon optimization tools now allow prediction of secondary structures with minimal free energy, facilitating the design of highly stable transcripts that maintain efficient translation [104]. Beyond structural stability, sequence engineering strategies are also employed to fine-tune immunogenicity. For example, reducing exposed uridine content within coding sequences decreases the likelihood of activating innate immune sensors such as TLR3, TLR7, TLR8, RIG-I, and MDA5, thereby minimizing unwanted immune activation [105]. More recently, advanced multitask deep convolutional neural networks have facilitated the development of models such as RiboNN, which predict translation efficiency by integrating features beyond the 5′ UTR, including codon composition and their spatial arrangement [106]. These features collectively influence ribosomal processivity and tRNA availability, both key determinants of translational output. Taken together, AI-guided models provide powerful new opportunities for the rational design of therapeutic mRNAs.

### Purification and quality control

While chemical and structural optimization of cmRNA significantly improves its therapeutic potential, purification remains a critical step in minimizing immunogenicity and enhancing consistency. *In vitro* transcription reactions often contain unwanted byproducts such as unincorporated nucleotides, truncated transcripts, enzymes, salts, uncapped RNA, and double-stranded RNA Among these, uncapped RNA and dsRNA are particularly problematic, as they strongly activate innate immune sensors and severely impair translation efficiency [51].

At the laboratory scale, cmRNA is often purified using isopropanol precipitation, enzymatic cleanup, or commercially available kits. For large-scale production, however, chromatographic methods are preferred because of their superior ability to remove immunostimulatory impurities and reliability in achieving batch-to-batch reproducibility [107]. Seminal work by Karikó et al. demonstrated that high performance liquid chromatography purification eliminates dsRNA contaminants, thereby abolishing type I interferon induction and increasing translation by up to several orders of magnitude, establishing this method as a cornerstone for therapeutic mRNA production [108].

Rigorous quality control is essential to guarantee product safety and efficacy. Unlike conventional biologics, mRNA therapeutics have distinct structural and functional requirements, and their critical quality attributes are still being refined. Key quality parameters include verifying complete DNA digestion and removal of truncated transcripts and dsRNA contaminants [109,110], as well as assessing sequence integrity, 5′ capping efficiency, poly(A) tail length and heterogeneity, and the absence of uncapped RNA [111,112]. Among these, capping yield is particularly important, as it directly influences translation efficiency and mRNA stability [113].

Recent advances in analytical chemistry have expanded the toolbox for mRNA characterization. Liquid chromatography-based methods allow detailed characterization of mRNA identity, structural integrity, and functional elements [114]. These methods are now applied to lot release testing, stability evaluation, and routine analysis of quality control attributes. By addressing the unique challenges of separating large, highly charged RNA molecules with dynamic secondary structures, these advances provide a path toward robust and standardized frameworks for therapeutic mRNA manufacturing.

## Methods for cmRNA delivery

Efficient intracellular delivery of mRNA is a prerequisite for successful transcript therapy. However, naked mRNA cannot freely cross the cell membrane due to its large molecular size, which is three orders of magnitude larger than passively diffusing molecules, and its strong negative charge, which repels the anionic cell membrane [33]. Moreover, naked mRNA is intrinsically vulnerable to degradation by 5ʹ- or 3ʹ-exonucleases and endonucleases [115]. Consequently, it is essential to design a delivery system that will carry the genetic information to the desired cells; not only for *in vitro* and *in vivo* studies but also to accomplish a broad and competent clinical application.

The ideal vector must enable high transfection efficiency while guarding the safety of the treatment; it must prevent triggering an immune response and preferably target a specific cell population. It must also overcome the cell membrane barrier without compromising membrane integrity. The lipid bilayer of cells, composed mainly of negatively charged phospholipids and regulated by ion pumps and channels, maintains a resting potential of −40 to −80 mV [116]. This electrochemical barrier hinders entry of mRNA unless specific mechanisms are employed.

**Fig. 3.**
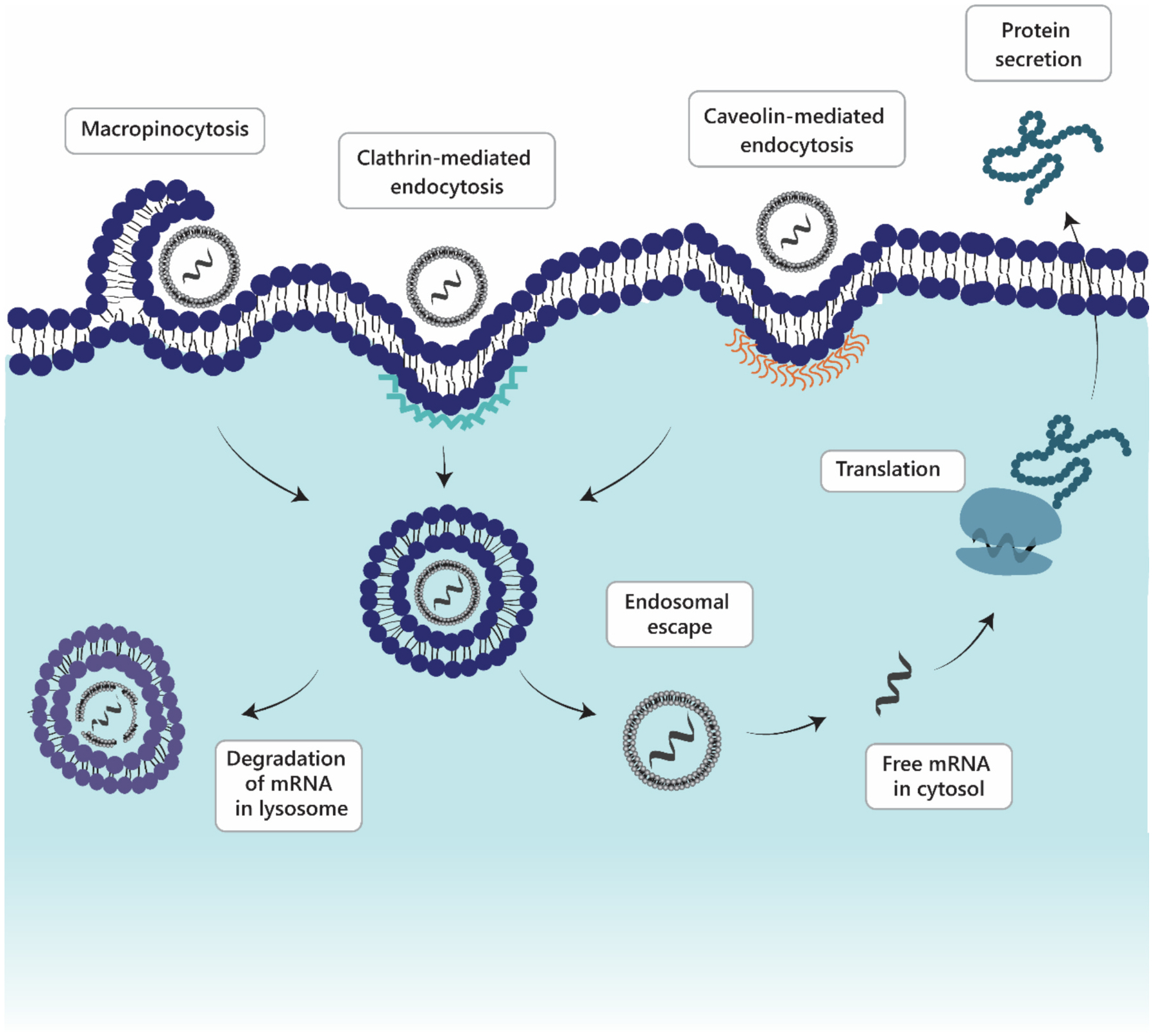
Cellular internalization and intracellular trafficking of mRNA-loaded vectors. The uptake of mRNA-vector complexes occurs through endocytic pathways. After internalization, successful delivery requires endosomal escape of the complex to release mRNA into the cytosol, where it can be translated by ribosomes into the therapeutic protein. If endosomal escape fails, the complex remains in the endocytic pathway and is ultimately trafficked to lysosomes, where both the vector and mRNA are degraded by hydrolytic enzymes.

When successful, internalization of cmRNA into the targeted cells and its translocation to the cytosol happens through multiple processes, including caveolae and clathrin-mediated endocytosis, micropinocytosis and macropinocytosis [117–119]. However, a major limitation is that most internalized mRNA remains trapped in the endosomal pathway, with only a fraction reaching the cytoplasm where translation occurs (Fig. 3). Overcoming this step is critical for enhancing protein expression and remains a major design focus for next-generation delivery platforms.

**Fig. 4.**
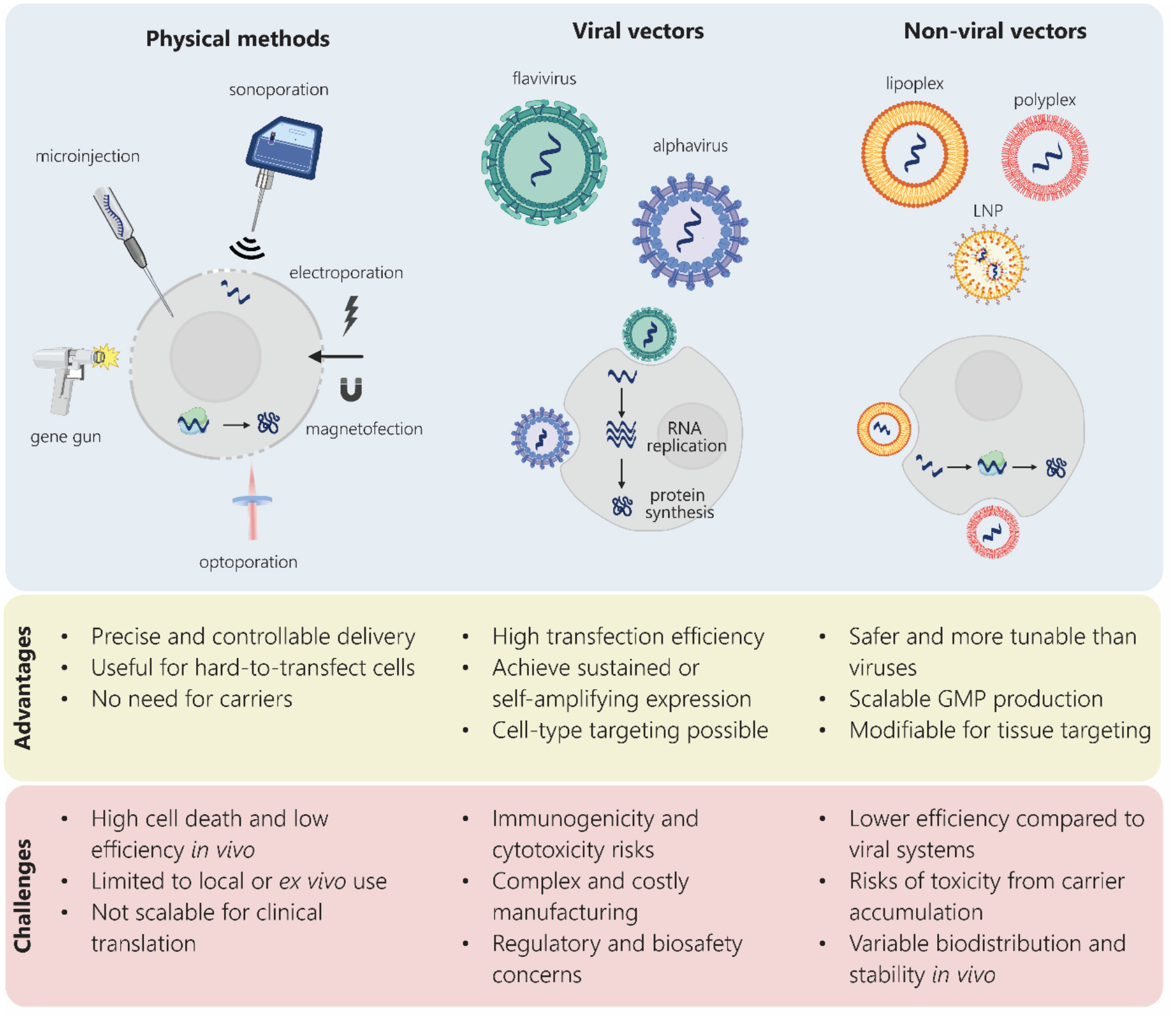
cmRNA delivery methods. Schematic illustration of the three principal strategies for cmRNA delivery: physical methods, viral vectors, and non-viral (chemical) carriers. Each approach employs distinct mechanisms to overcome cellular barriers and enable transcript uptake. The figure highlights the core advantages and challenges of these methods.

To address these challenges, three main strategies for cmRNA delivery have been established: physical methods, viral vectors, and nonviral (chemical) carriers (Fig. 4). Each approach offers distinct mechanisms, advantages, and limitations that guide their application in bone regeneration.

### Physical methods

Physical methods deliver nucleic acids into a cell by penetrating the cell membrane with force. The most studied methods include electroporation, sonoporation, magnetofection, optoporation, gene gun, and microinjection [120]. Electroporation, for instance, applies electrical pulses to the cell membrane shortly changing the membrane’s polarity so that DNA or mRNA can enter the cytoplasm. These methods are often highly efficient, meaning that transfected cells express high levels of the delivered gene, and can be used with any cell type. However, these physical techniques require invasive surgery and cause transient damage at the site of treatment. At the cellular level, they can disrupt the integrity of the cell membrane, traumatizing the cell and leading to its death [121]. Until now, few studies have reported the use of these techniques in bone tissue engineering to achieve mRNA delivery due to the high cost and inconvenience of *in vivo* delivery [120,122]. Therefore, in this review, we will focus on viral vectors and mainly non-viral vectors delivery systems.

### Viral vectors

Viruses are the most efficient gene delivery vectors, they have evolved a mechanism for delivering nucleic acids into cells, which makes them suitable for transcript therapy. The viral capsid offers protection of the encoded gene from extracellular ribonucleases [123].

The construction of viral vectors for mRNA delivery typically involves engineering replication-deficient or self-amplifying RNA viruses in which the viral structural or pathogenic genes are partially or completely replaced with the therapeutic transcript of interest [123]. In these systems, the viral genome is modified to retain only the essential nonstructural proteins required for replication and transcription, while eliminating genes responsible for viral assembly or pathogenicity. This ensures that the engineered vector can efficiently produce the target mRNA and drive protein translation in host cells, without generating infectious viral progeny [100]. In practice, construction involves cloning the therapeutic gene into a cDNA copy of the viral genome, followed by *in vitro* transcription to generate RNA replicons that can be packaged into viral-like particles.

In self-amplifying systems, once the RNA replicon enters the host cytoplasm, the retained nonstructural proteins form an RNA-dependent RNA polymerase complex that replicates the replicon. This process generates multiple copies of the therapeutic mRNA from a single input transcript, greatly amplifying the amount of protein that can be produced without additional vector input. Translation of the therapeutic gene proceeds directly from these replicated transcripts by host ribosomes [123].

Furthermore, specificity can be introduced by modifying the viral envelope glycoproteins to interact with particular cellular receptors, thereby enabling targeted delivery to desired cell types [124].

For mRNA recombinant protein production, positive-sense RNA viruses are the preferred vector systems. The most common available vector systems include the flavivirus and the alphavirus [125]. Comparative studies have shown that alphavirus enable high but short-lived expression due to cytotoxicity, while flavivirus support moderate yet prolonged expression [126]. These results suggested the relevance of alphavirus vectors for high-level short-term therapeutic expression and flavivirus when extended expression periods are required. Interestingly, flavivirus replicons can suppress type-I interferon production, which may benefit sustained, low-immunogenic protein expression [127].

Despite these advantages, viral vectors are limited by their immunogenicity, potential cytotoxicity, and high production costs. Safer alternatives, such as viral particles, are being explored. For example, Wang et al. used the nonstructural protein-1 (NS1) from Influenza A virus as a mRNA enhancer. The co-delivery of NS1 mRNA with mRNA of reporter genes significantly increased translation efficiency. The dual delivery of NS1 mRNA and BMP-2 mRNA to murine pluripotent stem cells promoted osteogenic differentiation and mineralization [128].

Still, the safety and regulatory challenges associated with viral systems restrict their clinical applicability in cmRNA therapies. As a result, attention has shifted toward non-viral delivery platforms.

### Non-viral vectors

Chemical-based vectors or non-viral vectors are gaining increasing attention given their diversity, and versatility. A wide variety of materials, including lipids, cationic polymers, peptides and gold nanoparticles can be used to transfer genetic information to target cells *in vivo*. They offer modularity, ease of production, reduced immunogenicity, and lower costs. Multiple chemical-based vectors have been investigated for bone regeneration.

#### Lipid-based vectors

**Fig. 5.**
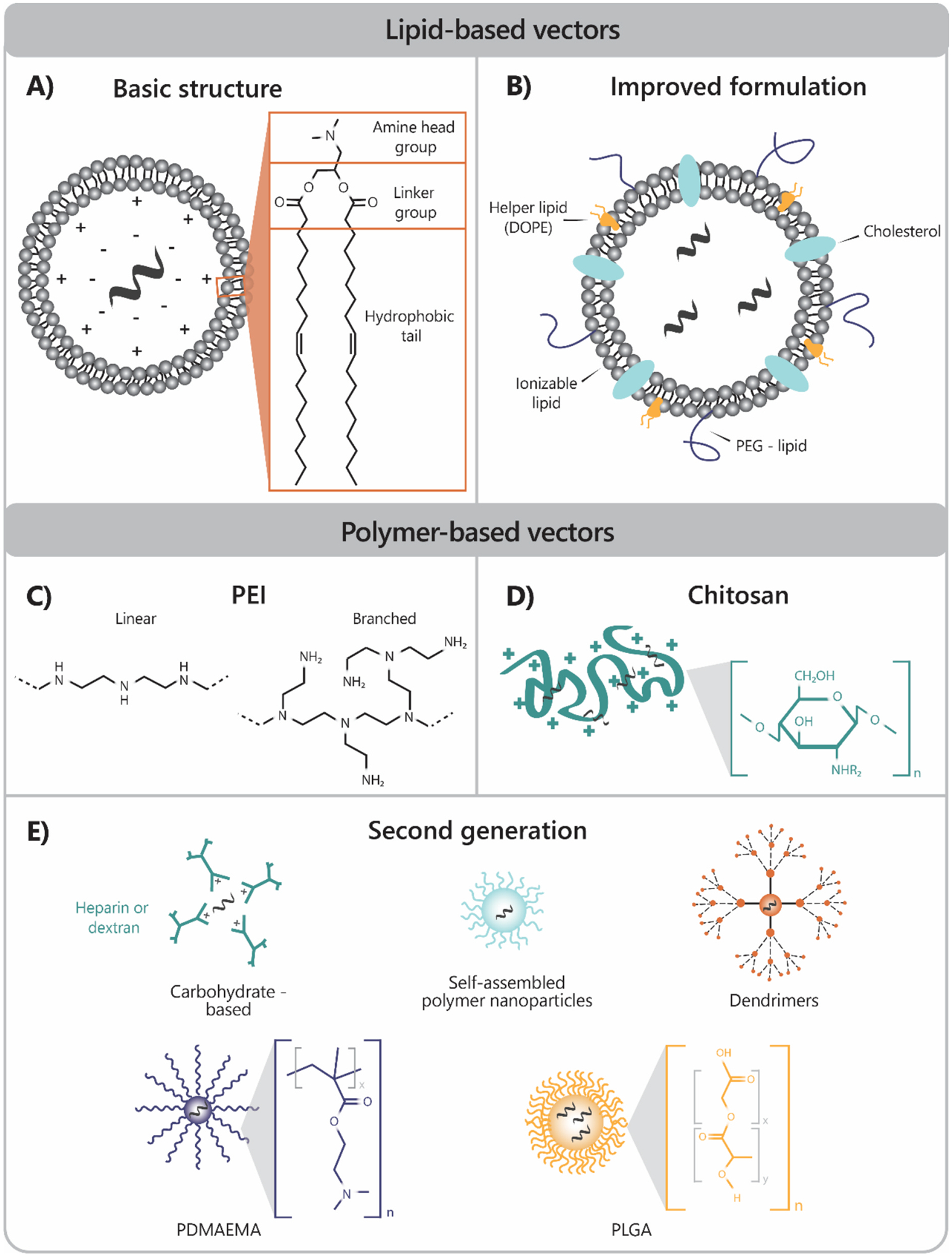
Common types of non-viral vectors for mRNA delivery: lipid-based and polymer-based systems. (A) Basic structure of lipid vectors includes an amine head group which is positively charged, a linker usually consisting of an ester, ether, carbamate, or amide and a hydrophobic tail. (B) Improved formulations of lipid vectors include the use of an ionizable or cationic lipid for mRNA encapsulation, a helper lipid that enhances its fusion to the cell membrane, cholesterol to stabilize the lipid bilayer and PEG lipid to improve colloidal stability, and reduce protein absorption. Typical polymer-based vectors include (C) PEI in its linear and branched configurations, and (D) chitosan. (E) Second-generation polymer vectors include carbohydrate-based polymers (e.g., heparin, dextran), self-assembled polymeric nanoparticles, dendrimers, PDMAEMA, and PLGA. Abbreviations: PEG, polyethylene glycol; PEI, polyethylenimine; PDMAEMA, poly[(2-dimethylamino)ethyl methacrylate]; PLGA, poly(lactic-co-glycolic acid).

Lipid vectors, or liposomes, are spherical vesicles consisting of one or more phospholipid bilayers surrounding an aqueous core that encloses the drug, in this case the mRNA. Cationic lipids interact electrostatically with negatively charged mRNA, forming lipid-mRNA complexes known as lipoplexes (Fig. 5A) [129]. The lipid head groups are usually amines or ammonium salts, while tails are aliphatic chains linked via ester, ether, or amide bonds [121]. These electrostatic and hydrophobic interactions drive the spontaneous encapsulation of mRNA, effectively shielding it from extracellular nucleases [130].

Lipoplexes not only protect mRNA from degradation but also promote cellular uptake through endocytosis [131]. Once inside the cell, their fusogenic properties enable endosomal escape, releasing the mRNA into the cytosol, which is a critical step to prevent lysosomal degradation [132]. Despite these advantages, first-generation lipoplexes suffer from important limitations, including short circulation times, non-specific tissue uptake, and cytotoxic effects such as pro-inflammatory and hepatotoxic responses upon systemic administration [121,133,134]. Their efficacy is further compromised by rapid clearance via the mononuclear phagocyte system and neutralization by anionic serum proteins [135,136].

To overcome these drawbacks, modern lipid-based vectors, particularly lipid nanoparticles (LNPs), have emerged as next-generation delivery systems and are now considered the gold standard in clinical mRNA therapeutics. Unlike lipoplexes, which are formed primarily by electrostatic interactions between cationic lipids and mRNA, LNPs are self-assembled nanostructures composed of ionizable lipids, helper lipids, cholesterol, and PEG-lipids. Each component plays a distinct role: ionizable lipids, often containing tertiary or quaternary amines, facilitate mRNA packing and endosomal release by becoming positively charged at acidic pH while remaining neutral at physiological pH, minimizing toxicity [137]; helper lipids (e.g., 1,2-dioleoyl-*sn*-glycero-3-phosphoethanolamine [DOPE] or 1,2-distearoyl-*sn*-glycero-3-phosphocholine [DSPC]) promote membrane fusion and enhance cellular uptake; cholesterol stabilizes the lipid bilayer by filling gaps between lipids; and PEG-lipids improve colloidal stability and reduce protein adsorption [138] (Fig. 5B). This architecture improves encapsulation efficiency, protects mRNA from degradation, and provides greater structural stability and controlled release, explaining the superior safety and efficacy of LNPs compared to conventional lipoplexes.

The clinical success of LNPs is exemplified by their use in COVID-19 mRNA vaccines, where they enabled efficient systemic delivery, high protein expression, and robust immune responses with an excellent safety profile [139]. Unlike vaccines for infectious diseases, LNPs for bone healing face several specific challenges, including the need for sustained mRNA stability and prolonged protein expression to match the longer healing times of skeletal tissues [18]. Additionally, effective targeting and retention of LNPs in the bone microenvironment is difficult due physiological barriers such as the blood-bone marrow barrier, and limited affinity of delivery vehicles for bone minerals. Strategies to address these challenges include optimization of dosing regimens to balance efficacy and immune activation, and incorporation of bone-targeting ligands (e.g., bisphosphonates) into LNP formulations to enhance localization and uptake while overcoming biological barriers [140].

The reproducibility and effectiveness of lipid-based formulations have facilitated the development of commercial reagents widely used for *in vitro* and *in vivo* mRNA delivery in bone regeneration [25]. For example, Balmayor et al. compared Lipofectamine2000, DreamFect^™^ Gold, and Dogtor for cmRNA delivery, reporting longer expression with the latter two [35]. Our group similarly assessed cmRNA transfection in human osteoblasts using lipid-based vectors (Lipofectamine 3000 and 3DFect) and a polymer-based vector (TransIT-X2), and found that lipid systems achieved significantly higher protein expression [118]. Beyond these commercial reagents, LNPs formulated with C12-EPE, DOPE, cholesterol, and PEG-lipids have been shown to effectively deliver BMP-2 and/or BMP-7 cmRNAs and promote bone regeneration *in vivo* [35,47,72]. Such vectors are most often combined with scaffolds to localize delivery; however, Yoon et al. recently developed bone-targeting ionizable lipids by incorporating a piperazine backbone with bisphosphonate moieties, enabling strong hydroxyapatite binding [141]. These piperazine-based LNPs accumulated efficiently in the bone microenvironment after systemic administration, underscoring the potential of rational lipid design for targeted cmRNA delivery to mineralized tissues.

At present, lipid-based vectors are considered the most effective mRNA delivery systems. However, not far away, the polymer-based vectors are also rising as potential tools for nucleic acid delivery.

#### Polymer-based vectors

Polymer-based vectors (polyplexes) are formed via electrostatic interactions between cationic polymers and nucleic acids. These vectors are chemically versatile, more stable than lipoplexes, and allow for extensive functionalization [25,32]. However, their clinical use is hampered by cytotoxicity mainly related to the poor clearance of large molecular weight polymers [131], and due to their difficult scale-up and manufacturing [142].

The earliest and most used cationic polymer is polyethylenimine (PEI). PEI has an abundance of positive charges derived from their secondary amines (see Fig. 5C), which are only protonated in the late endosome when the polyplex is exposed to lower pH. This feature enhances packing of the mRNA and its cellular uptake [143]. Its branching degree and molecular weight critically influence delivery efficacy and cytotoxicity, with high molecular weight (>25 kDa) PEI often associated with poor biocompatibility [136,144].

To reduce some of these problems, low-molecular-weight PEI modified with fatty acid chains has been used for mRNA delivery [145]. Elangovan et al. used 25 kDa branched PEI to deliver BMP-2 cmRNA, achieving high transfection efficiency and significant bone regeneration in calvarial defects with minimal cytotoxicity [46]. Khorsand et al. also reported osteogenic outcomes using PEI for BMP-2 and BMP-9 cmRNA delivery [41,146].

Lately, PEI has been replaced by other polymers to increase the biocompatibility of the delivery system. One of such alternatives is the natural polysaccharide chitosan. This polymer contains a high density of positive charges along its polymeric chain and can form complexes with the negatively charged mRNA [147] (Fig. 5D). While chitosan offers lower toxicity and improved biodegradability, it faces challenges such as poor solubility at physiological pH, instability, and inefficient delivery of the genetic cargo [148]. Modified forms like chitosan-PEI hybrids have shown promise in enhancing osteogenic differentiation and bone formation [149].

Second-generation polymers include poly[(2-dimethylamino)ethyl methacrylate](pDMAEMA), poly lactic-co-glycolic acid (PLGA)-based nanoparticles, self-assembled polymeric nanomicelles, carbohydrate-based polymers such as heparin or dextran, and dendrimers [129] (Fig. 5E). Self-assembled polymeric nanomicelles are made of block copolymers in which a polyamino acid block forms an inner core surrounded by an outer PEG ‘shell’ with mRNA complexed electrostatically in the center [150]. These polymers can be formed by simple synthesis using a wide array of monomers that provide multi-functionality to the delivery system. For instance, pH-responsive polymers can exploit pH drops in cellular compartments to improve mRNA delivery and endosomal escape [151].

Dendrimers are highly branched spherical macromolecules with a high population of primary, secondary, and tertiary amines. Their basic structure is usually well-defined in size and shape, it encompasses a central core, repetitive branching units, and terminal groups with adaptable surface functionalities [152]. Dendrimers promote gene delivery by facilitating nucleic acid uptake through their primary amines while the more sterically hindered tertiary amine groups promote endosomal escape [153]. The most common and promising dendrimer with higher levels of transfection is polyamidoamine (PAMAM). Beneficial aspects of PAMAM are the hydrophilic, biocompatible and nonimmunogenic properties [154]. In the upcoming years, we expect to observe a rise in dendrimers and polymer-based vectors with improved transfection efficiencies and biocompatibility for mRNA delivery, which may promote the clinical approval of these materials.

#### Hybrid lipid-polymer-based nanoparticles

**Fig. 6.**
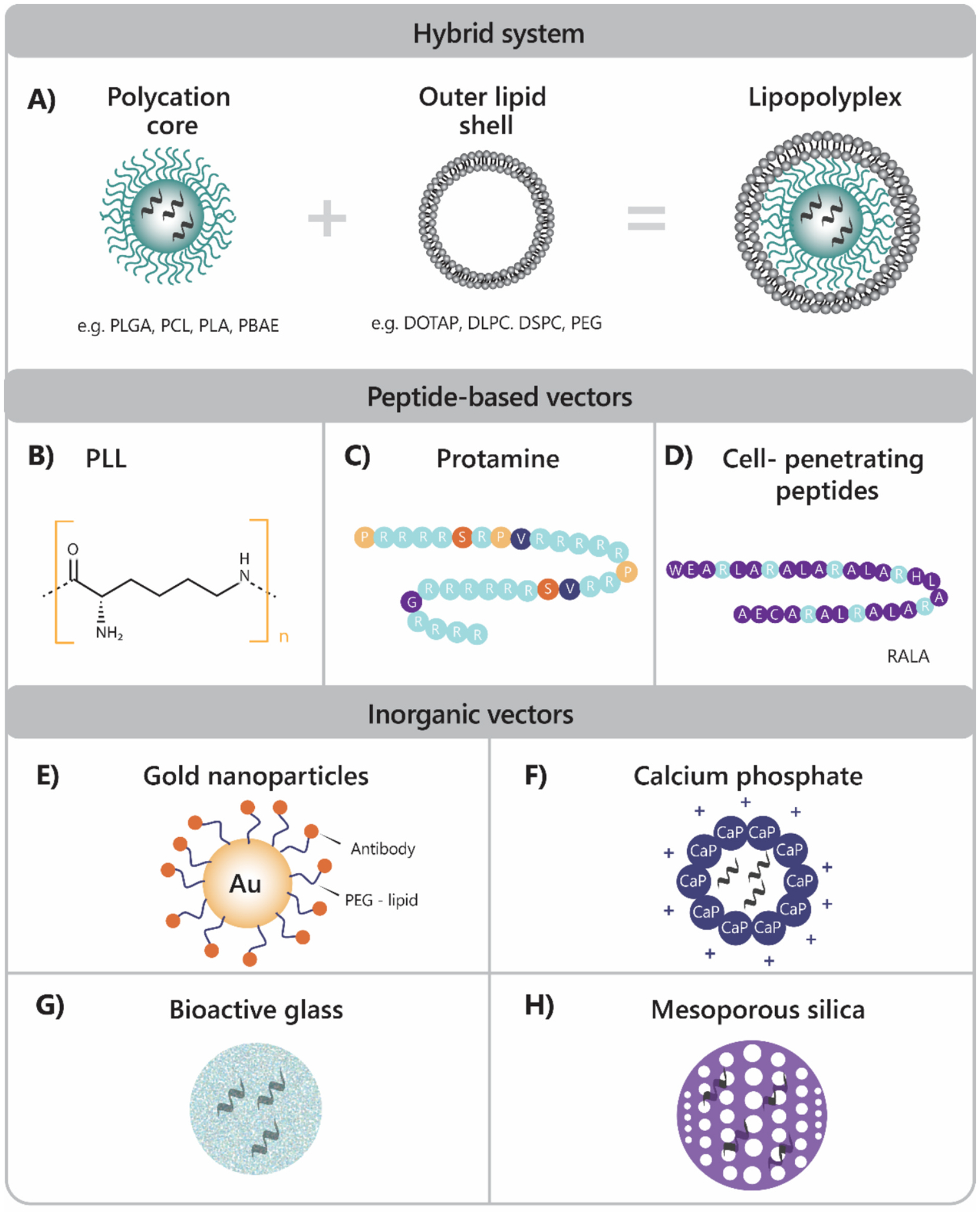
Non-viral vectors: hybrid systems, peptide-based vectors and inorganic vectors. (A) Schematic of a lipopolyplex, a hybrid nanoparticle composed of a polymer–mRNA complex encapsulated within a lipid shell. Peptide-based vectors include: (B) poly(L-lysine), (C) protamine, and (D) cell-penetrating peptides, such as the amphipathic arginine-rich peptide RALA. Inorganic nanoparticles explored for mRNA delivery include: (E) gold nanoparticles, (F) calcium phosphate, (G) bioactive glass, and (H) mesoporous silica. Abbreviations: PCL, polycaprolactone; PLA, polylactic acid; DOTAP, 1,2-dioleoyl-3-trimethylammonium-propane; PEG, polyethylene glycol.

Hybrid nanoparticles usually consist of a polycation-nucleic core with an outer layer shell consisting of lipids (Fig. 6A), and are commonly referred to as lipopolyplexes. Several polymers and lipids have been investigated to formulate stable nucleic acid lipid particles. Typical polymers employed are PLGA, polycaprolactone (PCL), polylactic acid (PLA), or their combinations, whereas the lipids used include 1,2-Dioleoyl-3-trimethylammonium propane (DOTAP), 1,2-dilauroyl-*sn*-glycero-3-phosphocholine (DLPC), 1,2-distearoyl-*sn*-glycero-3-phosphocholine (DSPC), lecithin and PEG-lipids.

Poly(β-amino esters) (PBAEs), a class of biodegradable polymers, have been extensively investigated for nucleic acid delivery and incorporated into hybrid systems with PEG-lipids to enhance colloidal stability [155]. In another study, a hybrid system consisting of a PLGA polymeric core and a lipid-like material (N1,N3,N5-tris(2-aminoethyl) benzene-1,3,5-tricarboxamide) enabled the functional delivery of mRNAs encoding firefly luciferase and enhanced green fluorescent protein across three cell lines [155]. Overall, hybrid lipid–polymer vectors provide thermodynamic stability and combine the complementary physicochemical properties of lipids and polymers, offering a promising platform for efficient nucleic acid delivery [156].

#### Peptide-based vectors

Cationic peptides are widely used vectors due to their capacity to interact and condensate negatively charged RNAs, simple synthesis, and versatility [157]. However, their tendency to form aggregates raises multiple limitations, including long-term stability, toxicity, poor transfection efficiency, and complicates the manufacturing process of the pharmaceutical product [158].

One of the earliest peptide-based delivery systems included poly(L-lysines) (PLLs) (Fig. 6B) [159]. PLLs on their own exhibit limited transfection capacity, are susceptible to proteolytic degradation and opsonization, and have demonstrated toxicity *in vivo* [160]. To overcome these limitations, PLLs have been widely functionalized with endosomal-disrupting agents and fusogenic peptides to enhance cytosolic delivery and endosomal escape. PLL functionalized with PEG has shown to reduce opsonization and increase circulation time [161]. Another example includes protamines (Fig. 6C) a family of arginine-rich proteins, which have shown efficient mRNA uptake by cells. A notable study developed antibody–protamine fusion proteins to enable antibody-mediated targeted siRNA delivery to HIV-infected or envelope-transfected cells [162].

A third class of peptide-based vectors are the cell-penetrating peptides. Cell-penetrating peptides are positively charged peptides of 5–30 amino acids in length that are capable of delivering a variety of biologically active payloads, such as peptides, proteins, and nucleotides inside cells [163]. The first cell-penetrating peptides to be described were the transactivator of transcription (TAT) and penetratine [164]. Later on, a cell-penetrating peptide with arginine-rich amphipathic RALA sequence repeats (Fig. 6D) was reported to deliver both eGFP and ovalbumin mRNA [165]. The study concluded that cell-penetrating peptide RALA outperformed the cationic lipid DOTAP and the fusogenic lipid DOPE. Nevertheless, disadvantages of this system are the short circulation half-life, due to the low stability in serum-containing medium, and relatively low transfection efficiency, given their low molecular-weight and inefficient gene condensation [166].

#### Inorganic vectors

One last category of non-viral vectors are the inorganic nanoparticles (NPs). Like polymeric vectors, the surface chemistry of inorganic NPs are highly tunable to enable cell targeting and to introduce functional groups [167]. Unique features of inorganic NPs are the high surface area to volume ratios for drug loading, and, depending on the nanoparticle type, the ease with which they can be imaged noninvasively due to their optical properties, thus allowing label-free monitoring of biodistribution [168]. The most relevant inorganic NPs for RNA delivery include gold (Fig. 6E), calcium phosphate (Fig. 6F), bioactive glass (Fig. 6G), and mesoporous silica (Fig. 6H) [169]. Despite the relevant application of these vectors, few groups have investigated them for mRNA delivery; most of the groups have used inorganic vectors for miRNA and siRNA transfer. In particular, bioactive glass is the only material examined for siRNA delivery to bone, with demonstrated cytocompatible transfections to preosteoblasts and marrow stromal cells [170].

Gold nanoparticles (GNPs) are promising platforms for nucleic acid delivery given their simple fabrication, functionalization and controllable *in vivo* toxicity and biodistribution by optimizing the particle size [171]. GNPs can be covalently attached to thiolated molecules, such as oligopeptides or thiolated PEG, which can further be linked to antibodies (Fig. 6E). PEG stabilizes the NP surface, prevents NP aggregation and reduces nonspecific protein adsorption on the NP surroundings [172]. Yeom et al. injected mRNA encoding Bcl-2-associated X protein (BAX), a proapoptotic factor, loaded on GNP-DNA conjugates into murine xenograft tumors. The delivered mRNA produced biologically functional BAX protein, and tumor growth was inhibited [173].

Inorganic vectors as well as peptides, lipids and polymers have come a long way and probably still need to further improve several aspects. However, data is accumulating to understand the mechanism of genetic delivery to the cytoplasmatic compartment of all these materials. Knowledge on cellular uptake is readily increasing as will their use in preclinical and clinical trials.

### Routes of cmRNA delivery to bone

In addition to selecting the appropriate cmRNA and delivery vector, it is equally important to consider how the mRNA complex reaches its target site. Delivering mRNA for fracture healing poses unique challenges compared with other tissues. Although healthy bone is well vascularized, the early fracture phase is marked by hematoma formation, which is initially avascular and hypoxic [174]. Repair then progresses through sequential yet overlapping stages: hematoma and inflammation, cartilage callus formation, vascular invasion, and ossification; each requiring precise spatial and temporal regulation of signaling factors [175]. Gene delivery systems must therefore remain effective within this dynamic microenvironment. The fracture site is also mechanically unstable, with interfragmentary movement and continuous remodeling that can hinder vector retention and homogeneous transfection. Importantly, systemic administration of osteoinductive factors carries the risk of severe off-target effects, such as ectopic bone formation in soft tissues, making localized delivery the preferred strategy [37]. This contrasts with tissues like liver or muscle, where systemic circulation can be harnessed for vector distribution. For bone, the central challenge is achieving durable, site-specific expression within a mechanically unstable and biologically heterogeneous environment while preventing unwanted transfection of surrounding tissues.

**Fig. 7.**
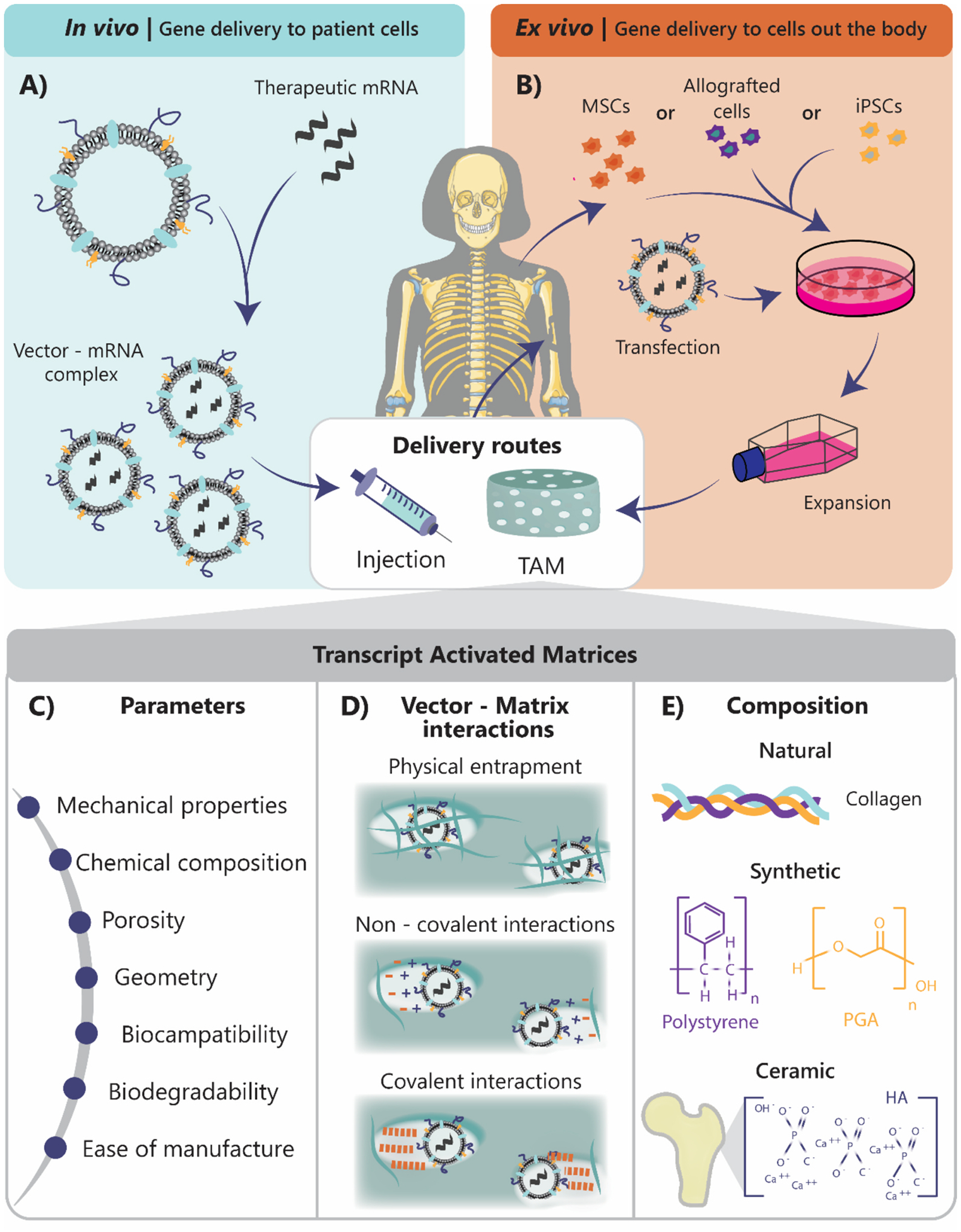
Routes of cmRNA transfer to bone through injections or scaffolds. (A) Via *in vivo* delivery, the transcript is directly applied into the patient. (B) Via ex vivo delivery, a given cell population is transfected outside the body and will be later introduced into the patient. (C) Design parameters to consider when designing TAMs. (D) Mechanisms of vector–matrix interactions. (E) Classification of biomaterials according to their composition.

Two principal strategies for gene delivery exist: (1) direct administration of the transcript into the patient, termed *in vivo* transfection (Fig. 7A), and (2) indirect administration via *ex vivo* transfection, in which cells are genetically modified outside the body before implantation (Fig. 7B). Both strategies can be implemented via injection or in combination with scaffolds to support bone regeneration [176]. The choice of delivery route significantly influences the duration of protein production and the need for repeated administrations to maintain therapeutic levels [131]. Given that accessing bone tissue often requires surgery, selecting a delivery strategy that minimizes invasiveness and maximizes efficacy is paramount.

### Direct injection

Initially, most investigators avoided direct injection of vectors containing osteogenic genes for bone regeneration as it has shown to promote a strong immune response, leading to little bone formation specially when using adenovirus vectors [177]. Nevertheless, recent studies have proven that it is a valid choice to introduce genetic material into bone. Multiple studies in small and large animals have reported fracture healing using recombinant adenoviral, retroviral or plasmid vectors with different genes [178], however none using mRNA.

A major drawback of using direct injection is a poor targeted delivery to specific cells. Typically, cells surrounding the tissue of interest may also be transfected leading to dispersion of the vector and therefore adverse side effects [179]. A study where a rabbit model of femoral defect was used to introduce marker genes by injection showed that higher expression of the transgene was observed in the musculature surrounding the defect, with significant expression also occurring in the gap scar and the cut ends of the bone [180].

### Transfection ex vivo

In *ex vivo* gene therapy, a specific cell population is isolated and expanded in culture. Then the cells are transfected *in vitro* with the gene of interest and implanted into the defect (see Fig. 7B). This approach offers precise control over the transfection process and allows selection of responsive cell types [178]. Cells used in this method must differentiate into osteoblasts under appropriate cues and secrete osteoinductive factors to stimulate bone repair via paracrine and autocrine pathways [176].

Initial studies using human bone marrow stem cells (BMSCs) transfected with BMP-encoding plasmids showed promise in rodents and pigs [181]. Subsequent research demonstrated that cells from skin, muscle, fat, and blood also display osteoinductive properties when transfected *ex vivo* using BMPs [182,183]. MSCs have been used successfully for healing defects in long bones, cranial structures, mandibles, and in spinal fusion, even in large animal models [178].

Despite showing promising results, *ex vivo* transfection is considered surgically invasive, technically complex, and expensive. Clinical translation requires adherence to GMP standards and entails two surgeries (cell harvesting and reimplantation), posing regulatory and logistical challenges [184].

To address this, alternatives include using allogeneic MSCs or induced pluripotent stem cells (iPSCs). While allogeneic MSCs from a universal donor could provide sufficient MSCs with osteogenic potential, they require immunosuppression, raising safety concerns [185]. iPSCs differentiated into MSCs offer a scalable, autologous alternative [186]. A further innovation is expedited *ex vivo* therapy, where tissue is harvested, modified, and reimplanted within a single surgical session [178]. Autologous muscle, adipose tissue, and marrow tissue have been used as they contain progenitor cells that can be genetically modified *in situ* [184]. Among these, adipose tissue is particularly attractive for this purpose due to its abundance and donor acceptance [187].

Whether applied *in vivo* or *ex vivo*, gene delivery often employs biodegradable scaffolds to retain cells or complexes at the defect site. In some cases, direct injection suffices. The next section focuses on scaffold-based systems, particularly for *in vivo* applications.

### Transcript activated matrices

Bone healing using mRNA therapy relies on inducing cellular differentiation and proliferation. Achieving spatial and temporal control over this process is essential due to the complex 3D architecture of bone. Transcript-activated matrices (TAMs) are biomaterial scaffolds engineered to deliver mRNA complexes in a sustained and localized manner [188]. This platform provides a three-dimensional template for cell infiltration and tissue formation, while maintaining a safe and efficient release of therapeutic mRNA.

One advantage of TAMs over direct injections is that they can retain the gene complexes within the matrix for longer durations [189]. This feature is particularly beneficial to enhance the lengthy processes blood vessel formation and bone matrix deposition [190]. In addition, TAMs also offer spatial confinement of the delivered transgenes [191]. For these reasons, TAMs are the preferred delivery route for mRNA-based therapy for bone regeneration.

#### Design parameters

TAMs must meet essential design criteria including: porosity, geometry, chemical composition, mechanical strength, biocompatibility, and biodegradability (Fig. 7C) [192]. A highly homogeneous interconnected pore network will promote efficient transport of metabolites and nutrients [193]. Composition and degradation profile critically influence cmRNA loading and release kinetics, while surface chemistry affects cell migration and adhesion. It is desirable that the scaffold contains binding sites for specific cell populations [192]. Functionalization with specific ligands, such as high mobility group box 1 (HMGB1), can promote recruitment of osteoblasts and endothelial cells during endochondral ossification [194].

Mechanically, TAMs must withstand physiological loads and preserve structural integrity. With advanced techniques like 3D bioprinting, scaffold geometry can be customized to the defect, enabling functional bone regeneration that preserves native shape and size [195].

Finally, the selected biomaterial must meet clinical requirements such as a suitable degradation rate, integration with the surrounding native bone without triggering an immune response. and easy to manufacture under GMP standards [196].

#### Entrapment mechanism

In order to achieve a prolonged and confined gene delivery, it is important to understand the physico-chemical interactions between the TAM and the vectors carrying the mRNA. There are three main strategies for incorporating mRNA vectors into TAMs (Fig. 7D) [30]:

- Physical entrapment: Vectors are embedded within the scaffold’s porous structure. Release depends on matrix degradation and diffusion of the cmRNA complexes [197]. Careful consideration is needed during scaffold fabrication to avoid vector damage from heat, solvents, or shear stress [198]. An example is fabricating scaffolds using a gas foaming process to encapsulate complexes into a porous engineering scaffold. The foaming method generates highly porous scaffolds with interconnected pore structures that encapsulate nucleic acids without the use of organic solvents.
- Non-covalent interactions: Vectors are adsorbed onto scaffold surfaces through electrostatic, hydrophobic, or Van der Waals forces [199]. This method enables post-fabrication loading without degrading the mRNA. Natural and synthetic materials such as collagen, ceramics, PLGA, and PCL can be used to fabricate these platforms [70,72,200]. A drawback of this method is the release of adsorbed mRNA due to enzymatic reactions and competitive protein adsorption by the use of media containing serum proteins [30].
- Covalent interaction: chemical immobilization of vectors to the scaffold offers tighter control over release kinetics. Specific interactions are introduced through complementary functional groups on the vector and the scaffold. Strategies include biotin-streptavidin conjugation or amine/thiol coupling reactions [201].

#### Composition

TAMs are ideally composed of biodegradable and biocompatible materials that can be resorbed upon implantation into osseous defects. Biomaterials can be classified in natural, synthetic or ceramic (Fig. 7E) [202].

- Natural materials (e.g., collagen, fibrin): These support cell differentiation and matrix deposition but often lack mechanical strength. Collagen I-based scaffolds have been successfully used in load-bearing and non-load-bearing defects [71,72]. Similarly, fibrin gels have been used to load BMP-2 cmRNA in rat femoral defects showing improved bone healing as early as 2 weeks after implantation [35].
- Synthetic materials (e.g., polystyrene or PLGA): They offer tunable mechanical properties and are suitable for large-scale production, but may generate toxic degradation byproducts, lack bioactivity or spontaneous integration into surrounding tissues [30].
- Ceramics (e.g., hydroxyapatite, β-tricalcium phosphate): These provide excellent stiffness and biocompatibility, though brittleness is a limitation [196]. An example of a ceramic material used for delivery of cmRNA to bone was a micro–macro biphasic calcium phosphate (MBCP) TAM, which showed efficient cmRNA release [203].

It is also possible to develop composite scaffolds that offer the advantages of combing the mechanical strength of ceramics with the biological activity of naturally derived materials or with the tunability of synthetic materials. For example, Utzinger et al. developed EPO cmRNA entrapped in calcium phosphate cement (CPC)/PLGA micro-spheres that could be used for bone regeneration applications [70].

Hydrogels, both natural or synthetic 3D networks of polymers that swell and retain large amounts of water, have brought a lot of excitement in the tissue engineering community [204]. For example, it is possible to control the level of biodegradability, mechanical properties, injectability, and microstructure, by modifying their polymer to water ratio, or their degree of polymerization [205]. In addition, they can be engineered to respond to specific stimuli, such as enzymatic activity, pH, temperature, light, and electricity [206]. While PEG-based hydrogels offer electrostatic neutrality, cationic hydrogels allow prolonged mRNA retention via electrostatic interactions [207,208]. Nevertheless, cationic polymers activate apoptotic signaling pathways and are cytotoxic despite the enhanced transfection outcomes.

## *In vivo* and *in vitro* cmRNA studies for bone regeneration

Over the last 20 years, the field of mRNA therapeutics has seen remarkable growth, driven by advancements in synthetic biology, delivery systems, and chemical modifications that enhance transcript stability and translational efficiency. Numerous biotechnology companies, including CureVac, BioNTech, Moderna, eTheRNA, Ethris, Trilink, and Factor Bioscience, have emerged as leaders in mRNA-based technology [209]. In parallel, major pharmaceutical companies such as Shire, Novartis, AstraZeneca, Takeda, and Sanofi-Pasteur have also entered the field. There are multiple clinical trials registered that are using mRNA therapy, mostly for infectious diseases, cancer treatment and immunotherapy applications [209]. By contrast, the application of mRNA for musculoskeletal repair remains in the early preclinical phase, with most studies restricted to proof-of-concept investigations in small animal models or *in vitro* models (Table 2) [210].

### BMPs cmRNA

As BMPs are the dominant bone stimulating factors, they have been the preferred molecules to study the bone regeneration potential of cmRNAs. The first proof-of-concept study by Elangovan et al. [46] demonstrated that BMP-2 cmRNA complexed with branched PEI enhanced osteoblastic differentiation of BMSCs *in vitro*, while *in vivo* implantation in collagen scaffolds increased bone volume in rat calvarial defects. Importantly, pseudouridine/5mC modifications avoided interferon activation and outperformed unmodified mRNA and pDNA.

Building on this, Badieyan et al. [72] showed that vacuum-dried BMP-2 cmRNA-collagen sponges achieved nearly 100 % transfection efficiency, low cytotoxicity, and protein expression lasting up to 11 days. TAMs regenerated bone in a rat femoral defect and remained stable at room temperature for six months, highlighting translational promise.

In a comparative study, Khorsand et al. [41] evaluated BMP-2 versus BMP-9 cmRNAs. While both constructs enhanced bone formation *in vivo*, BMP-9 induced significantly higher alkaline phosphatase activity, calcium deposition, and trabecular connectivity in calvarial defects. A subsequent study by the same group used perforated collagen membranes as delivery platforms for BMP-9 pDNA or cmRNA, confirming that BMP-9 cmRNA outperformed its plasmid counterpart in promoting new bone formation [146].

Balmayor et al. [35] optimized BMP-2 cmRNA with ARCA, poly(A) tail, and 5mC/2tU substitutions, delivering it via cationic lipids (C12-EPE with DOPE and cholesterol). In MSCs and human adipose cells, the construct yielded high BMP-2 expression and osteogenic marker upregulation. Remarkably, rats treated with only 2.5 μg BMP-2 cmRNA/defect achieved significant femoral healing, a tenfold dose reduction compared to Elangovan’s earlier work [46]. Zhang et al. [71] further improved outcomes by incorporating TISU sequences, removing uORFs/AU-rich elements, and introducing 5-iodo pyrimidines, resulting in strong protein yield, minimal inflammation, and durable bone formation over eight weeks. Ferizi et al. [78] demonstrated that UTR engineering, particularly using cytochrome *b*-245 alpha chain elements, further enhanced mRNA stability and osteogenic activity.

More recently, De la Vega et al. [45] addressed critical-sized segmental defects in rat femurs, considered among the most difficult to repair. Optimized BMP-2 cmRNA at doses ≥ 25 μg localized efficiently, drove robust bone healing, and restored mechanical strength. Unlike recombinant BMP-2 protein, cmRNA treatment avoided large callus formation and accelerated remodeling. Using a site in which osteogenesis occurs via intramembranous ossification, Surisaeng et al. [211] compared direct *in vivo* BMP-2 mRNA delivery via lipid nanoparticles with *ex vivo* transfected BMSCs, both of which improved calvarial healing, though *ex vivo* strategies yielded superior regeneration.

Together, these studies position chemically modified BMP mRNAs as powerful osteoinductive agents capable of driving bone repair at lower doses and with fewer side effects than recombinant proteins. Key lessons learned include: (i) chemical modifications (e.g., pseudouridine, 5mC, 5-iodo pyrimidines) are critical for stability, immune evasion, and efficient translation; (ii) delivery format strongly influences efficacy, with TAMs, lipoplexes, and *ex vivo* modified cells offering distinct advantages; and (iii) newer designs incorporating optimized UTRs and transcript architecture enable longer protein expression and stronger osteogenesis.

### Combinatorial approaches

Because vascularization is essential for bone repair, VEGF cmRNA has been combined with BMP-2 to potentiate both osteogenesis and angiogenesis. Geng et al. [48] co-delivered BMP-2 and VEGF-A cmRNAs into exogenous BMSCs embedded in collagen sponges. This dual approach significantly enhanced osteogenic gene expression, mineralization, and bone regeneration in critical-size calvarial defects, outperforming single-transcript or protein-only treatments.

Similarly, our group has demonstrated the accelerated bone-forming potential of co-delivering BMP-2 and BMP-7 cmRNAs in mice [47]. This dual-cmRNA strategy enhanced osteogenic gene expression and mineralization more effectively than either single-transcript or sequential delivery. Mechanistically, BMP-2 primarily stimulates the early differentiation of hMSCs into osteoblasts, while BMP-7 supports later stages of osteogenesis, providing complementary effects across the osteogenic cascade. In addition, simultaneous delivery of BMP-2 and BMP-7 facilitates the formation of BMP-2/−7 heterodimers, which possess higher receptor-binding affinity and stronger downstream signaling than their homodimer counterparts [212].

Collectively these studies highlight the advantages of integrating two or more cmRNAs. By simultaneously targeting complementary biological processes such as angiogenesis and osteogenesis, or by combining factors with osteoinductive functions at distinct stages, dual-transcript delivery can achieve more robust and coordinated tissue repair. Future work should explore broader combinations to harness synergistic effects and further optimize regenerative outcomes.

### Transcription factors

Bone-targeted lipid nanoparticles encoding m^7^G-methylated Runx2 mRNA have shown promise in senile osteoporosis models [49]. By promoting osteoblast differentiation and bone formation in aged mice, this study introduces the concept of transcription factor-based cmRNA therapeutics as a highly potent approach. Unlike growth factors like BMP-2, transcription factors such as Runx2 directly modulate lineage commitment and cellular reprogramming, offering new possibilities for hard-to-heal fractures in aging populations.

### Extracellular matrix proteins

Pan et al. explored using cmRNA encoding amelogenin [50], a key extracellular matrix protein involved in the formation of the hard outer layer of teeth, for periodontal regeneration. The cmRNA was delivered via a thermosensitive, self-assembling hydrogel and promoted osteogenesis and cementogenesis in human periodontal ligament stem cells. This approach, although targeting alveolar bone rather than load-bearing skeletal elements, broadens the therapeutic scope of mRNA-based bone regeneration, particularly in dentistry and maxillofacial surgery.

Notably, these studies highlight the versatility of mRNA therapeutics across different skeletal sites. While long bones typically regenerate through endochondral ossification, calvarial and alveolar bone healing relies on intramembranous ossification. These findings underscore the potential of mRNA-based therapies to support bone regeneration irrespective of anatomical location or ossification mechanism.

### Scaffold strategies

Novelty in cmRNA therapeutics also implicates development of new scaffolds and biomaterials. Comparative studies between fibrin gels and MBCP granules [203], and the use of PLGA microspheres within self-hardening calcium phosphate cements [70], highlight the importance of matching scaffold properties with therapeutic goals such as sustained release, biodegradability, and cell migration for *in vivo* efficacy. Emerging strategies have also extended transcript therapy beyond *de novo* tissue formation. Fayed el al. [213] explored the use of cmRNA coatings to enhance the osteointegration of titanium implants. Researchers tested three biomaterials (PDLLA, fibrin, and fibrinogen) as carriers for BMP2 cmRNA on titanium surfaces. Fibrinogen coatings demonstrated the highest transfection efficiency, prolonged protein expression, and superior osteogenic activity *in vitro*. While this does not involve direct tissue replacement, it addresses a long-standing challenge in orthopedics. Recently, granular hydrogels composed of hyaluronic acid and collagen have been introduced as 3D-printable carriers for BMP-7 cmRNA, supporting efficient transfection, high cell viability, and robust osteogenic differentiation of hMSCs [214]. This approach highlights the potential of patient-specific, bioactive scaffolds to replace autologous bone grafts and protein delivery in regenerative medicine.

## Potential clinical applications

Protein therapy and gene therapy have an optimistic but controversial future in the field of bone regeneration. translating these approaches into clinical practice remains challenging due to high costs, safety concerns, and regulatory hurdles. For example, only a few recombinant proteins have been FDA-approved for bone repair, and gene therapy protocols for musculoskeletal regeneration are scarce [215,216]. Traditional gene therapy is often viewed as risky and difficult to regulate, especially since musculoskeletal conditions are typically non-lethal, making the cost-benefit ratio harder to justify [26,217].

cmRNA offers a safer and more cost-effective alternative, avoiding genomic integration and benefiting from streamlined manufacturing. This is particularly relevant for bone regeneration, where reducing treatment costs is essential to improve the quality of life for millions affected by fractures. Nevertheless, significant work remains. Comprehensive pharmacology, toxicology, and biodistribution studies are needed, along with proof-of-efficacy in large-animal models as a prerequisite for clinical trials. Although the success of mRNA vaccines highlights the strong translational potential of this technology, its application in bone regeneration is still at a critical preclinical stage.

**Fig. 8.**
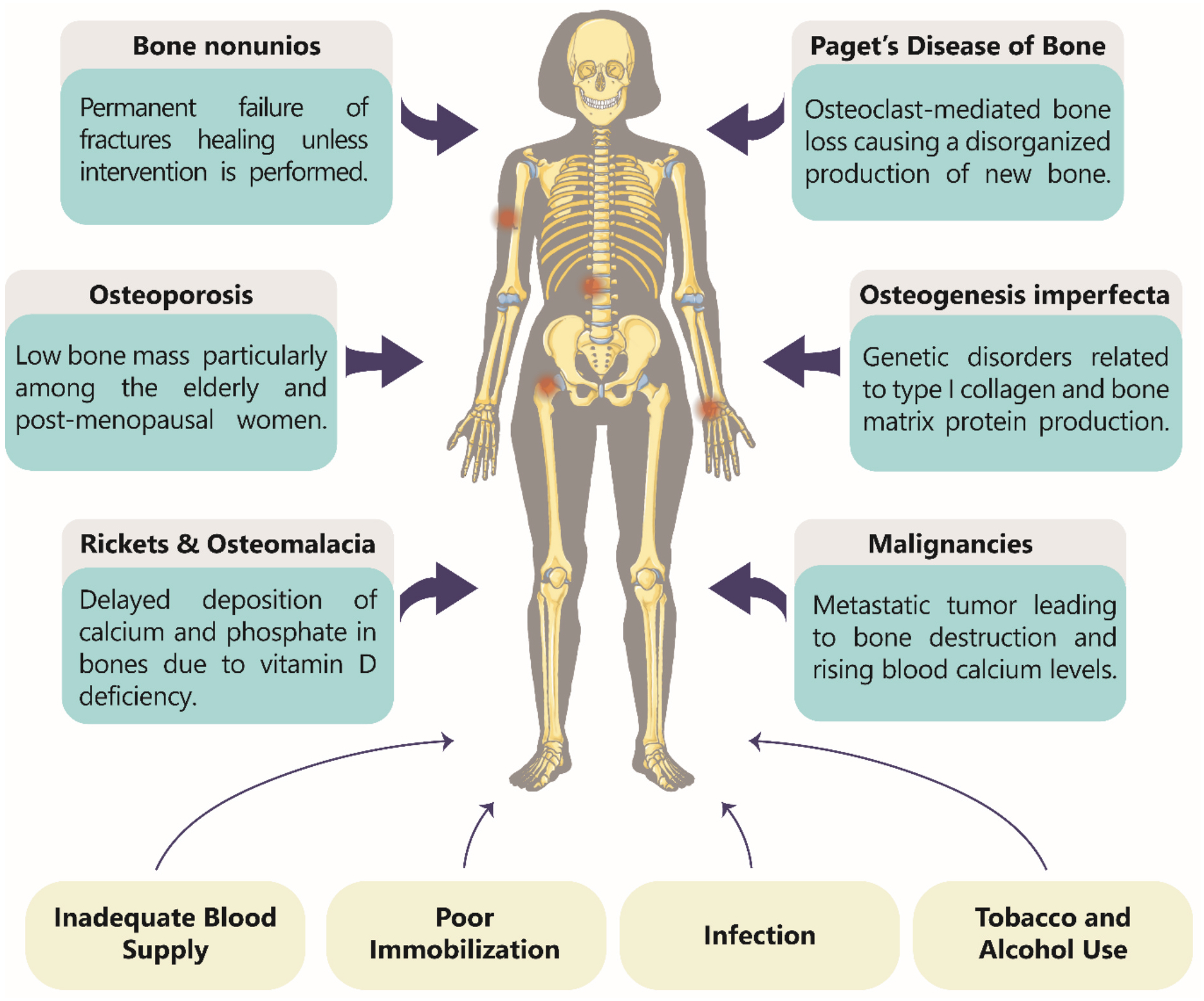
Potential clinical applications of chemically modified mRNAs encoding osteogenic factors for bone regeneration. This illustration highlights therapeutic opportunities for cmRNA-based interventions in conditions such as osteoporosis, bone defects associated with cancer or rare skeletal disorders.

It is important to recall that cmRNA technology does not only apply to those patients who suffer severe accidents resulting in nonunion fractures, but also to those ones who suffer bone malignancies (e.g. osteosarcoma), degenerative and genetic diseases, becoming extremely prone to have a non-self-healing fracture. Some examples of these pathologies are explained in Fig. 8. This may also be relevant for patients with diabetes, infections, poor fracture immobilization, smokers, and fractures with inadequate blood supply.

In addition to broad orthopedic applications, cmRNA therapeutics hold strong promise in the context of precision medicine. The inherent flexibility of cmRNA design and rapid adaptation of therapeutic constructs enables the production of personalized therapies that can be tailored to the patient’s specific genetic background, disease profile, or fracture microenvironment. This is especially advantageous for patients with heterogeneous or complex conditions, such as those with combined metabolic and degenerative bone diseases, where a “one-size-fits-all” treatment is unlikely to be effective. For example, precision strategies could involve delivering cmRNAs encoding osteogenic factors in combination with angiogenic or immunomodulatory proteins, adjusted according to biomarkers of impaired healing identified in a specific patient. This modularity could also support the development of combination therapies tailored to distinct phases of fracture repair or to patient-specific comorbidities, such as diabetes-associated delayed healing or cancer-related bone loss.

cmRNA synthesis can be rapidly adapted to produce customized batches with relatively minor changes in the manufacturing pipeline. This agility positions cmRNA as a practical tool for personalized orthopedic therapies, potentially accelerating the move from generalized interventions toward precision-guided bone regeneration strategies.

## Affordability

Data regarding the cost of a fracture is variable from study to study as differing estimates result from the method used (economic modeling or real-world data), the type of fracture, whether calculating direct costs or including indirect costs, the population studied and so on. Nevertheless, all studies agree in the huge and increasing economic burden that fractures represent [218]. In the U.S., about 1.6 million bone grafts are performed annually, totaling ~$244 billion in healthcare costs [10,219]. Protein-based therapies, although effective, remain costly. BMP-7 treatment for non-union fractures has raised concerns, with an average cost exceeding £21,000 per case. While some analyses report favorable cost offsets (e.g., rhBMP-2 in open tibial fractures), affordability remains a barrier to widespread use.

cmRNA offers a promising alternative with comparable efficacy and reduced production costs. Its manufacturing process avoids reliance on animal-derived materials, ensures batch consistency, and is scalable to GMP-grade production [220]. However, improvements in the large-scale production of reagents like ribonucleoside triphosphates and T7 polymerase are still needed to meet clinical demand [39]. Although detailed cost analyses of cmRNA for bone fracture treatment are limited, experts estimate that GMP-grade mRNA production for clinical applications is 5–10 times more cost-effective than recombinant protein therapeutics, which require complex and specialized purification processes [209]. Insights from COVID-19 mRNA vaccine production further support this cost advantage. For instance, while Moderna and Pfizer charged affluent nations $14.70 to $23.50 per dose, large-scale manufacturing costs were estimated at just $0.54 to $0.98 per dose [221,222]. Similarly, the projected cost for mRNA-based HIV vaccines, factoring in capital, personnel, and materials, was approximately $2.85 per dose [221]. Techno-economic analyses further show that RNA vaccine production can be rapidly scaled at relatively small bioreactor volumes, with costs largely determined by RNA dose per vial, underscoring the feasibility of meeting high global demand [223].

The cost advantage of mRNA technologies becomes even more evident when compared to conventional DNA-based gene therapies, which range from $500,000 to $1 million per treatment. These high costs stem primarily from the challenges of scaling up viral vector production and achieving a robust downstream purification process to ensure clinical-grade material. Here, we described non-viral mRNA delivery, specifically using lipid vectors, as an affordable option to AAV-based gene therapies. Non-viral vectors enable scalable and efficient manufacturing, substantially lowering production costs and simplifying purification processes.

## Potential risks of cmRNA therapeutics

The worldwide deployment of COVID-19 mRNA vaccines has offered valuable data regarding the safety of mRNA-based therapies. In general, these vaccines have shown a strong safety record, with most side effects being mild to moderate in nature, commonly including local injection site reactions (pain, swelling, redness) and transient flu-like symptoms (fatigue, muscle aches, fever, chills) [224]. The incidence and severity of these adverse events depend on factors such as administration route and dosage; for example, intradermal injections are generally more reactogenic than intramuscular injections [98,225,226]. In contrast, localized delivery via scaffold-based systems may help mitigate systemic immune activation, though this approach requires further validation.

Unlike vaccines, cmRNA-based protein replacement or regenerative therapies often require substantially higher doses to achieve therapeutic efficacy, with concentrations at least 15 times higher [98,99]. Such higher concentrations of both cmRNA molecules and their delivery vehicles may trigger innate immune pathways and/or cytotoxicity. Unmodified or insufficiently modified mRNAs can activate endosomal Toll-like receptors (TLR3, TLR7/8) or cytosolic RNA sensors (RIG-I, MDA5), leading to type I interferon production and inflammation [227,228]. At the same time, excessive mRNA can overwhelm the cellular translation machinery, causing an accumulation of unfolded or misfolded proteins in the endoplasmic reticulum, triggering stress and apoptosis [229]. Similarly, cationic lipids and polymers used for encapsulation can destabilize cellular membranes, induce oxidative stress, or disrupt mitochondrial function, contributing to off-target toxicity [136,230]. These mechanisms highlight the need for rational design of both cmRNA chemistry and vector formulations.

For bone regeneration specifically, a single intervention would represent the ideal therapeutic approach to reduce invasiveness: a TAM loaded with cmRNA placed at the fracture site that enables protein production over a period of days, rather than requiring multiple administrations. Under these conditions, the risk of strong T cell–mediated immune rejection is unlikely, particularly if the cmRNA encodes a human protein. The transient nature of cmRNA expression, combined with the immunomodulatory properties of both cmRNA modifications and modern LNP formulations, makes it more probable that the therapy will avoid eliciting a strong T cell response and instead promote a state of local immune tolerance. This localized and time-limited mode of action is a key advantage particularly beneficial for promoting bone regeneration.

Safety monitoring, however, has to consider that adverse effects may not manifest immediately but could appear over time, even after a single administration. Locally, the risks include chronic inflammation at the implantation site. Furthermore, if the expressed protein levels are too high (such as with osteogenic or angiogenic factors), it could lead to ectopic calcification, abnormal bone remodeling, or vascular malformations. Systemic risks are expected to be lower with localized delivery; however, potential long-term concerns such as vector-related toxicity, immune tolerance alterations, or delayed tissue responses should not be overlooked. Additionally, if cmRNA therapeutics are ever combined with other drugs or systemic interventions, there remains the possibility of synergistic immunotoxic or metabolic side effects that warrant careful evaluation.

To mitigate these risks, clinical studies should employ conservative dose-escalation regimens, careful monitoring of circulating antibodies against both cmRNA and encoded proteins, and functional assays to evaluate organ-specific toxicity. Equally important is the assessment of inter-individual variation in metabolism and immune responsiveness, which may guide personalized dosing strategies. Since adverse effects may emerge only after repeated or long-term dosing, extended follow-up periods and combination safety studies will be crucial for the successful clinical translation of cmRNA therapeutics in this field.

## Conclusions

Non-healing bone defects represent a major clinical challenge with limited therapeutic options. mRNA-based protein expression offers a compelling approach for bone regeneration, combining the benefits of gene therapy with improved safety and regulatory profiles. This technology avoids genomic integration and enables controlled, transient protein production, which is ideal for the time-sensitive nature of bone healing.

mRNA is inherently unstable, requires protection from immune recognition, and must be efficiently delivered to target cells. Advances in structural modifications of cmRNAs and the development of delivery vectors, particularly LNPs, polymers, and hybrid systems, are progressively addressing these issues. Another hurdle is the need for optimized administration strategies, while *in vivo* delivery is quite straightforward, it exhibits limitations related to poor control over gene expression, undesired biodistribution and lack of target specificity. Conversely, *ex vivo* gene delivery allows better control of targeting cells and expression levels at the expense of complex procedures, high costs, and risk of immune rejection. Regardless of the selected route, the incorporation of a TAM greatly assists bone regeneration as it provides support that improves the mechanical stability of the insert and serves as a template for cell infiltration. It also allows host cells to take up de cmRNA in a sustained and transient fashion.

Looking ahead, successful clinical translation will require robust pharmacology, toxicology, and biodistribution studies, alongside demonstration of efficacy in large-animal models. Moreover, progress in industrialization, including scalable manufacturing, standardized quality control, and cost-effective production, will be critical to make cmRNA-based therapies accessible for widespread clinical use. In this regard, the rapid development and deployment of COVID-19 mRNA vaccines has set a precedent, offering valuable lessons to accelerate the translation of cmRNA-based therapies for bone regeneration and beyond.

Overall, cmRNA therapeutics hold strong potential to advance bone regeneration, offering a safer and more versatile alternative to protein and gene therapies. With continued innovation in molecular design, delivery systems, and translational frameworks, cmRNA may evolve into a clinically viable and economically sustainable treatment option for patients with traumatic or pathological bone defects.

## Figures and Tables

**Table 1:** Function of osteogenic growth factors and their use in transcript therapy.

Protein	Other names	Function in bone regeneration	cmRNA application for bone	Ref.
**BMP-2**	BMP-2A, BDA2	Stimulates bone and cartilage formation. Induces differentiation of osteoblasts.	Promotes bone regeneration of calvarial and femoral defects in rats.	[46,35,45]
**BMP-7**	OP-1	Promotes bone formation by inducing osteoblast differentiation, stimulating extracellular matrix production, and enhancing endochondral ossification.	Enhances ectopic *de novo* bone formation in combination with BMP-2 cmRNA in subcutaneous implantation in mice.	[47]
**BMP-9**	GDF-2	Induces differentiation of osteoblasts from MSCs.	Induces bone regeneration in calvarial defects in rats.	[41]
**VEGF**	VPF	Mediates angiogenesis. Stimulates differentiation and metabolism of preosteoblasts. Increases the recruitment, survival and activity of osteoclasts.	Enhances osteogenesis through the simultaneous stimulation of angiogenesis in an *in vivo* rat calvarial defect model.	[48]
**Runx2**	AML3, CBFA1, OSF2, PEA2aA	Promotes osteoblastic differentiation and skeletal morphogenesis. Acts as a scaffold for nucleic acids and regulatory factors involved in skeletal gene expression	Promotes bone repair and formation in the bone defect mouse model and senile osteoporosis mice.	[49]
**AMELX**	AMG, AIH1, AI1E	Induces biomineralization during tooth enamel development.	Results in sustained expression of AMELX in human periodontal ligament stem cells and promotes their osteogenesis and cementogenesis. Regenerates bone and periodontal ligament tissues in periodontal defects of rats.	[50]

**Abbreviations**: AMELX, AMG and AIH1, Amelogenin,; AML3, Acute Myeloid Leukemia 3 Protein; BMPs and BDA2, bone morphogenetic proteins; CBDA1, Core-Binding Factor Subunit Alpha-1; GDF-2, growth differentiation factor 2; MSC, mesenchymal stem cell; OP-1, osteogenic protein 1; OSF2, Osteoblast-Specific Transcription Factor 2; PEA2Aa, Polyomavirus Enhancer-Binding Protein 2 Alpha A Subunit; Runx2, Runt-Related Transcription Factor 2; VEGF, vascular endothelial growth factor; VPF, vascular permeability factor.

**Table 2:** Summary of cmRNA publications for bone regeneration.

Gene	Modifications	Vector	Material	Model	Dose	Time	Ref.
*In vivo*							
**BMP-2**	– ARCA – Poly(A) tail – 25 %: 5mC and 2TU – 100 %: Ψ and 5mC	Branched PEI	Collagen	Rat calvarial bone defect	25 μg/ defect	4 weeks	[46]
**BMP-2**	– 5′ cap – Poly(A) tail – 25 % 5mC and 2TU	Proprietary cationic lipid	Collagen	Rat non-critical femoral defect	2.5 μg/ defect	2 weeks	[72]
**BMP-2 or BMP-9**	– ARCA – Poly(A) tail – 100 %: Ψ and 5mC	PEI	Collagen	Rat critical size calvarial bone defect	50 μg/ defect	4 weeks	[41]
**BMP-9**	– ARCA – Poly(A) tail – 100 %: Ψ and 5mC	Branched PEI	Collagen	Rat critical size calvarial bone defect	10 μg/ defect	4 weeks	[146]
**BMP-2**	– ARCA – Poly(A) tail – 25 %: 5mC and 2TU	C12-EPE	Fibrin	Rat femoral defect	2.5 μg/ defect	2 weeks	[35]
**BMP-2**	– 5′ cap – Poly(A) tail – TISU element – 35 % 5 IU, 7.5 % 5IC – 25 % 5mC and 2TU	Cationic lipid with DPPC and cholesterol	Collagen	Rat critical sized femoral bone defect	1.25 or 5 μg/ defect	8 weeks	[71]
**BMP-2 and VEGF-A**	– Poly(A) tail – 100 % m1Ψ	Lipofectamine MessengerMAX^™^ Reagent	Collagen	Rat critical size calvarial bone defect	2.5 μg/ defect	4 and 12 weeks	[48]
**BMP-2**	– 5′ cap – Poly(A) tail – TISU element – 35 % 5 IU, 7.5 % 5IC – 25 % 5mC and 2TU	Cationic lipid with DPPC and cholesterol	Collagen	Critical-sized femoral defects	5, 10, 25, and 50 μg/ defect	4, 6, and 8 weeks	[45]
**BMP-2, PDGF- BB, FGF-2**	– Full uridine replacement with m1Ψ	Lipofectamine 2000 and LNPs	Custom-made 3D porous scaffolds	Rat model with calvarial defects	*In vivo*: 1.5, 5, or 15 μg/ scaffold *Ex vivo:* 2 μg mRNA/ well	4 weeks	[211]
**BMP-2 and/or BMP-7**	– For BMP-2: – 35 % 5 IU, 7.5 % 5IC – For BMP-7: 25 % 2TU, 25 % 5Mc – Poly(A) tail	Cationic lipid with DPPC and cholesterol	Collagen-nanohydroxyapatite	Subcutaneous ectopic bone formation model in mouse	10 μg of cmRNA per TAM	4 and 8 weeks	[47]
**Amelogenin**	– Poly-(A) tail – Full uridine replacement with m1Ψ	Lipofectamine MessengerMAX^™^ Reagent	PLA-PEG-PLA hydrogel	Critical-size alveolar defects in rats	2 ng/2.5 μL messengerMax + 5.5 μL of hydrogel	4 and 8 weeks	[50]
**Runx2**	m7G RNA	Zoledronic acid-DSPC, cholesterol, and DMG-PEG-2000 LNPs	n.a. (delivered via tail injection)	Senile osteoporotic mouse	1 mg/kg, once a week for 4 weeks	4 weeks	[49]
*In vitro*							
**BMP-2**	– ARCA – Poly(A) tail – 25 % 5mC and 2TU	DreamFect Gold	– Fibrin gel – MBCP granules	Osteogenic stimulation of BMSCs	1 μg/ gel or granule	2 and 3 weeks	[203]
**EPO**	– Poly(A) tail – 5mC and 2TU	Lipoplexes	CPC/PLGA microspheres	Murine fibroblasts/myoblasts	5 pg/ cell	4 days	[70]
**BMP-2**	– ARCA – Poly(A) tail – 1.89 mM 5mC and 1.89 mM 2TU – CYBA UTR	DreamFect Gold		Osteogenic stimulation of hAMSCs	20 pg/ cell	3 weeks	[78]
**BMP-2**	– Poly(A) tail −35 % 5 IU, 7.5 % 5IC –ARCA	cationic lipidoid with DPPC, cholesterol helper lipids, and DMG-PEG 2000	cmRNA coating titanium implants	C2C12 cells	62.5, 125, 250, 500 ng/ disk	7 weeks	[213]
**BMP-7**	– 25 % 2TU, 25 % 5Mc – Poly(A) tail	Lipofectamine MessengerMAX^™^	Hyaluronic acid and collagen hydrogels	Human BMSCs	1.56 pg/cell	4 weeks	[214]

**Abbreviations**: ARCA, anti-reverse cap analog; 5mC, 5-methylcytidine5′-triphosphate; 2TU, 2-thiouridine-5′-triphosphate; Ψ, pseudouridine-5′triphosphate; m1Ψ, N1-methylpseudouridine; PEI, polyethylenimine; CYBA, cytochrome b-245 alpha chain; DPPC, dipalmitoyl phosphatidylcholine; 1,2-dimyristoyl-rac-glycero-3-methylpolyoxyethylene PEGylated lipid, DMG-PEG 2000; PDGF-BB, Platelet-derived growth factor subunits B; FGF-2 Fibroblast growth factor 2; hAMSCs, human adipose mesenchymal stem cells; MBCP, micro–macro biphasic calcium phosphate; BMSC, bone marrow stem cell; EPO, erythropoietin; CPC, calcium phosphate cement; PLGA, poly (lactic-co-glycolic acid); 5 IU, 5-iodo-uridine; 5IC, 5-iodo-cytidine; DPPC, 1,2-dipalmitoyl-sn-glycero3-phosphocholine.

## Data Availability

Data will be made available on request.
